# Analysis of hyperforin (St. John’s wort) action at TRPC6 channel leads to the development of a new class of antidepressant drugs

**DOI:** 10.1038/s41380-022-01804-3

**Published:** 2022-10-12

**Authors:** Yamina El Hamdaoui, Fang Zheng, Nikolas Fritz, Lian Ye, Mai Anh Tran, Kevin Schwickert, Tanja Schirmeister, Albert Braeuning, Dajana Lichtenstein, Ute A. Hellmich, Dorothee Weikert, Markus Heinrich, Giulia Treccani, Michael K. E. Schäfer, Gabriel Nowak, Bernd Nürnberg, Christian Alzheimer, Christian P. Müller, Kristina Friedland

**Affiliations:** 1 Pharmacology & Toxicology, Institute for Pharmaceutical and Biomedical Sciences, Johannes-Gutenberg Universität Mainz (JGU), Mainz, Germany; 2 Institute of Physiology and Pathophysiology, Friedrich-Alexander-Universität Erlangen-Nürnberg (FAU), Erlangen, Germany; 3 Institute of Organic Chemistry and Macromolecular Chemistry, Faculty of Chemistry and Earth Science, Friedrich Schiller University Jena, Jena, Germany; 4 Biochemistry, Department of Chemistry, Johannes-Gutenberg Universität Mainz, Mainz, Germany; 5 Department of Food Safety, German Federal Institute for Risk Assessment, Max-Dohrn-Str. 8-10, 10589 Berlin, Germany; 6 Cluster of Excellence “Balance of the Microverse”, Friedrich-Schiller-Uniersität Jena, Jena, Germany; 7 Center for Biomolecular Magnetic Resonance, Goethe-University, Frankfurt, Germany; 8 Department of Chemistry and Pharmacy, Friedrich-Alexander-Universität Erlangen-Nürnberg (FAU), Erlangen, Germany; 9 Department of Psychiatry and Psychotherapy, University Medical Center of the Johannes Gutenberg-University Mainz, Mainz, Germany; 10 Institute of Anatomy, University Medical Center of the Johannes Gutenberg-University Mainz, Mainz, Germany; 11 Department of Anesthesiology, University Medical Center of the Johannes Gutenberg-University Mainz, Langenbeckstr. 1 (Bld. 505), 55131 Mainz, Germany; 12 Department of Pharmacobiology, Jagiellonian University Medical College, Krakow, Poland; 13 Department of Pharmacology, Experimental Therapy & Toxicology, Eberhard-Karls-University of Tübingen, Tübingen, Germany; 14 Department of Psychiatry and Psychotherapy, Friedrich-Alexander-Universität Erlangen-Nürnberg (FAU), Erlangen, Germany; 15 Centre for Drug Research, Universiti Sains Malaysia, 11800 Minden, Penang Malaysia

**Keywords:** Depression, Neuroscience

## Abstract

St. John’s wort is an herb, long used in folk medicine for the treatment of mild depression. Its antidepressant constituent, hyperforin, has properties such as chemical instability and induction of drug-drug interactions that preclude its use for individual pharmacotherapies. Here we identify the transient receptor potential canonical 6 channel (TRPC6) as a druggable target to control anxious and depressive behavior and as a requirement for hyperforin antidepressant action. We demonstrate that TRPC6 deficiency in mice not only results in anxious and depressive behavior, but also reduces excitability of hippocampal CA1 pyramidal neurons and dentate gyrus granule cells. Using electrophysiology and targeted mutagenesis, we show that hyperforin activates the channel via a specific binding motif at TRPC6. We performed an analysis of hyperforin action to develop a new antidepressant drug that uses the same TRPC6 target mechanism for its antidepressant action. We synthesized the hyperforin analog Hyp13, which shows similar binding to TRPC6 and recapitulates TRPC6-dependent anxiolytic and antidepressant effects in mice. Hyp13 does not activate pregnan-X-receptor (*PXR*) and thereby loses the potential to induce drug-drug interactions. This may provide a new approach to develop better treatments for depression, since depression remains one of the most treatment-resistant mental disorders, warranting the development of effective drugs based on naturally occurring compounds.

## Introduction

Depression is a severe mental disorder with a lifetime prevalence of more than 10% [1]. In addition to depressed mood, symptoms such as loss of interest, anhedonia, anxiety, feelings of worthlessness, weight loss, insomnia, and concentration deficits occur [2, 3]. Several different antidepressants such as selective serotonin uptake inhibitors (SSRI) or tricyclic drugs are used in daily medical practice to treat depressed patients [2]. However, patients suffer from several long-lasting and adherence-reducing side effects such as weight gain or sexual dysfunction or show partial- or non-response to classical antidepressants.

Patients with mild to moderate depression welcome plant-derived antidepressants such as St. John’s wort due to fewer side effects than commonly prescribed synthetic antidepressants. The herbal antidepressant St. John’s wort has been used for centuries to treat mild to moderate depression [4–6]. Hyperforin, the major antidepressant constituent, is an acylated bicyclic phloroglucinol derivative with little structural and functional resemblances with any known therapeutically used antidepressant. The antidepressant mechanism of action of hyperforin is intensively under discussion [7]. In heterologous expression systems and non-neuronal cells, hyperforin has been proposed to act as a protonophore in outer and inner cell membranes, thereby impeding uptake and vesicular storage of various neurotransmitters including monoamines [8]. By contrast, we have shown that hyperforin activates transient receptor potential channel 6 (TRPC6) channels and hypothesized that this effect is essential for its antidepressant profile [9, 10]. TRPC6 is a member of the TRP superfamily. TRP channels are homo- and/ or heterotetramers of subunits containing six transmembrane segments (S1–S6) and cytoplasmic N- and C-terminal tails [11]. S5, S6, and the connecting pore loop form the cation-conducting pore. S1–S4 and the cytoplasmic N and C termini are important for channel gating and the interaction with ligands or proteins [12–16]. The human TRPC subfamily comprises seven members, TRPC1 to TRPC7 [15, 17]. For different TRPC channels including TRPC6, cryo-EM structures were recently published, e.g. [18–20]. However, highly flexible regions in the C terminus which might contribute to conformational changes during activation were not resolved [19–22]. Importantly, hyperforin only activates TRPC6 channels and not the closely related TRPC3 and TRPC7 channels [9].

TRPC6 channels are expressed in several brain areas relevant for depression such as the dentate gyrus, where channel expression is particularly prominent, as well as in cortical regions [23–27]. We and others have shown that TRPC6 channels are involved in synaptic plasticity changes ranging from dendritic growth, spine morphology changes and increase in excitatory synapses [25, 28]. Hyperforin acts as a brain-derived neurotrophic factor (BDNF) mimetic in hippocampal neurons modifying dendritic spine morphology *via* TRPC6 channels [24]. However, hyperforin is not stable when exposed to light and oxygen [29]. It also induces drug–drug interactions due to potent activation of the nuclear receptor *PXR* (NR1I2), a key transcriptional regulator of genes involved in drug metabolism and transport [30]. These features limit its clinical application and require further developments of therapeutically applicable antidepressants.

Using a combination of behavioral analyses, electrophysiology, site-directed mutagenesis, and chemical synthesis, here we demonstrate that TRPC6 knockout (KO) mice show depressive and anxious behavior. This is paralleled by reduced activity of granule cells in the dentate gyrus and pyramidal neurons in the CA1 region of the hippocampus as a potential depressogenic mechanism. We identify an amino acid stretch within the cytoplasmic C-terminus essential for the direct interaction of hyperforin with TRPC6. Based on these insights, we synthesized the hyperforin analog Hyp13, which is a chemically simplified phloroglucinol. We show that Hyp13 has antidepressant effects that are TRPC6-dependent. Furthermore, Hyp13 does neither activate *PXR* not induce *CYP3A4*. Our findings highlight the crucial role of TRPC6 channels in depression and anxiety. Therefore, we suggest TRPC6 as a novel drug target for antidepressant therapy.

## Results

### Genetic TRPC6 deficiency induces depressive and anxious behavior

**Fig. 1:**
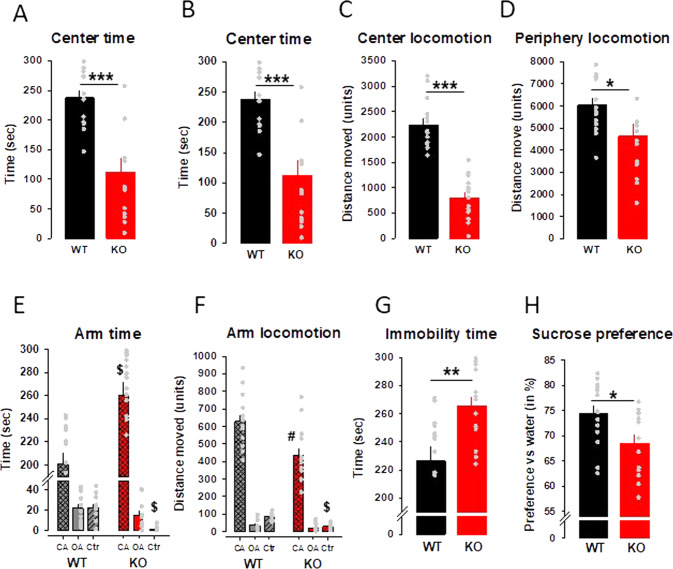
TRPC6 KO mice exhibit anxious and depressive behavior compared to wild-type (WT) mice. **A**–**D** Behavior in the open field test. **E**, **F** Behavior in the elevated plus maze test (OA open arm, CA closed arm, Ctr center). **G** Behavior in the forced swim test. **H** Sucrose preference test. Data are expressed as means ± s.e.m. (*n* = 14 per group; *p < 0.05, **^/#^*P* < 0.001, ***^/$^*P* < 0.001 vs WT).

In order to test an involvement of TRPC6 in depression and anxiety, we use a TRPC6 KO mouse model [31]. Male TRPC6 KO mice display anxious behavior in the open field test and the elevated plus maze test. In the open field test, TRPC6 KO mice show significant reduced center time (Fig. 1A), reduced number of center entries time (Fig. 1B), diminished center locomotion (Fig. 1C) and periphery locomotion time (Fig. 1D) compared to wild-type (WT) mice. In the elevated plus maze (EPM) test, TRPC6 KO mice spend significantly more time in the closed arm (CA) compared to WT animals (Fig. 1E). The arm locomotion in the closed arm and the center of the maze are significantly reduced in the TRPC6 KO mice (Fig. 1F). In the forced swim test (FST), the TRPC6 KO mice are characterized by a significantly increased immobility time (Fig. 1G). In the sucrose preference test (SPT), reflecting anhedonia, TRPC6 KO mice show a reduced sucrose preference compared to the WT animals time (Fig. 1H). Altogether these findings show a clear depressive/anxious phenotype in mice lacking the TRPC6.

### TRPC6 deficiency results in reduced hippocampal excitability

**Fig. 2:**
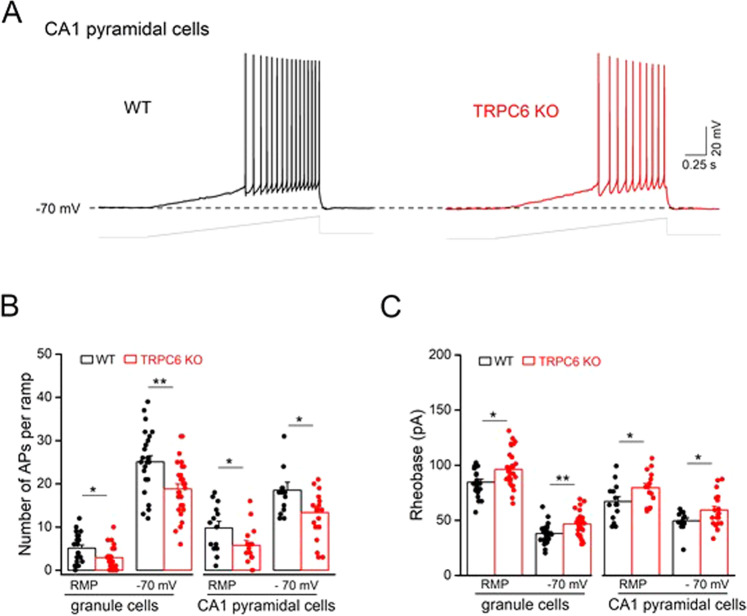
Reduced hippocampal cell excitability in TRPC6 KO mice. Whole-cell current-clamp recordings were performed from hippocampal CA1 pyramidal cells and dentate gyrus granule cells in brain slice preparation. Action potentials (APs) were evoked with a depolarizing ramp pulse from 0 to 100 pA for 2 s from resting membrane potential (RMP) or −70 mV (adjusted by current injection). **A** Voltage traces from CA pyramidal cells from a WT and a TRPC6 KO slice illustrate the evoked APs. The dashed line indicates −70 mV and the gray lines below show the ramp protocol. Histograms summarize the number of APs per ramp (**B**) and the rheobase (the minimal current necessary to elicit first AP; **C** in WT and TRPC6 KO hippocampal cells. *p < 0.05; **p < 0.01.

The hippocampus is a key structure for emotional regulation which may give rise to depression and anxiety disorders [32–36]. In order to identify a possible neuronal correlate for depression- and anxiety-like behavior in TRPC6 KO mice, we investigate DG and CA1 neuronal excitability in acute hippocampal slices from WT and KO mice using whole-cell current-clamp recordings. Comparable resting membrane potential (RMP) is observed in WT and TPRC6 KO CA1 pyramidal cells (WT, −72.9 ± 0.8 mV, *n* = 14 from 9 mice; TRPC6-KO, −74.3 ± 0.9 mV, *n* = 18 from 6 mice, *p* = 0.26) and DG granule cells (WT, −86.0 ± 0.5 mV, *n* = 25 from 15 mice; TRPC6-KO, −86.3 ± 0.5 mV, *n* = 29 from 8 mice, *p* = 0.70). Compared to their WT counterparts, TRPC6 KO granule cells have lower membrane input resistance (*R*_N;_ at −70 mV: WT, 325.0 ± 10.9 MΩ; TRPC6 KO, 288.9 ± 10.8 MΩ; *p* = 0.02), while there is no significant difference between WT and mutant CA1 pyramidal cells (WT, 194.1 ± 9.4 MΩ; TRPC6 KO, 204.4 ± 12.3 MΩ; *p* = 0.53). To probe cellular firing properties, we evoke action potentials (APs) with a depolarizing current ramp (0–100 pA over 2 s) starting from the neurons´ RMP or, for better comparison between groups, from a membrane potential of −70 mV, which is adjusted by current injection. As illustrated by representative voltage traces depicted in Fig. 2A that are obtained from a WT and a TRPC6 KO CA1 pyramidal cell, genetic disruption of *trpc6* reduces the firing propensity, such that less APs are fired per ramp depolarization when compared to the WT neuron. Both CA1 pyramidal cells and DG granule cells from TRPC6 KO mice exhibit a significantly mitigated discharge pattern independent of whether firing is evoked from rest or from −70 mV (Fig. 2B). Vice versa, rheobase, the minimal current necessary to induce the first AP during ramp depolarization is significantly enhanced in DG granule cells and CA1 neurons from TRPC6 KO mice compared to WT mice (Fig. 2C). These data show that constitutive loss of TRPC6 function engenders lasting alterations of basic firing properties of DG and CA1 neurons.

### TRPC6 deficiency abrogates hyperforin-induced increase in DG cell excitability

**Fig. 3:**
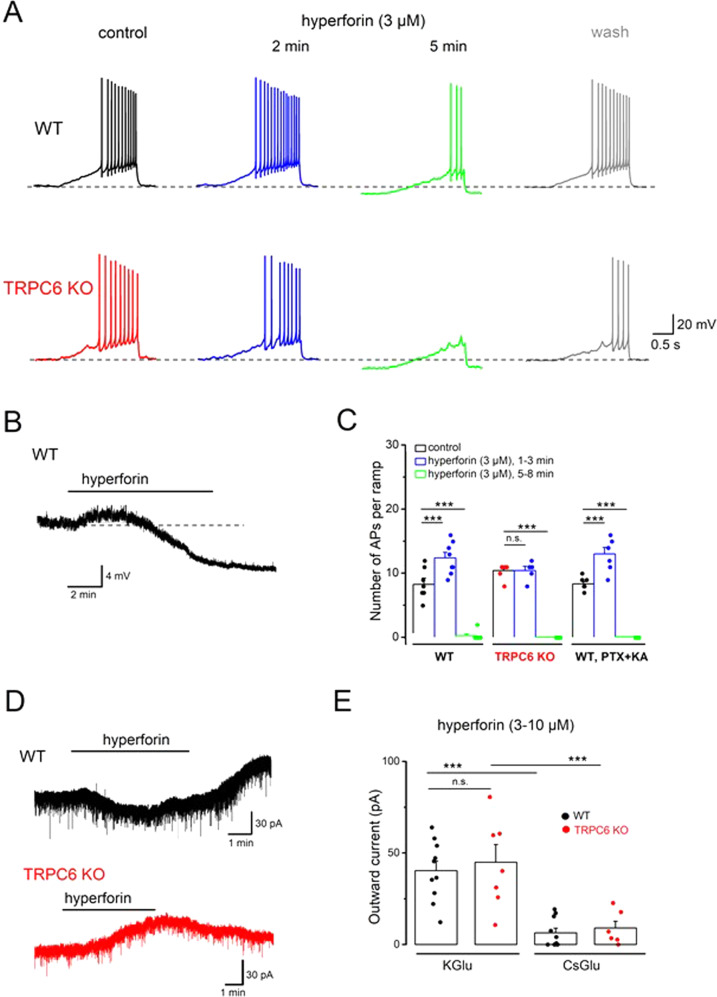
Loss of hyperforin-induced excitation in dentate gyrus granule cells of TRPC6 KO mice. **A–C** Whole-cell current-clamp recording of granule cells show effects of hyperforin (3 µM) on evoked APs (**A**, **C**) and on membrane potential (**B**). Dashed lines indicate −70 mV, depolarizing ramp was 0–70 pA for WT granule cell and 0–100 pA for TRPC6 KO cell. The hyperforin-induced biphasic response in wt slices was preserved after blocking fast synaptic transmission with kynurenic acid (KA) and picrotoxin (PTX) (**C**). **D**, **E** Voltage-clamp recordings (held at −70 mV) illustrate loss of hyperforin-induced initial inward current in neurons from TRPC6 KO mice (**D**). The remaining outward current involves K^+^ channels, as indicated by the loss of current with CsGlu-filled pipette (**E**). ***p < 0.001.

We next examine the effect of hyperforin on DG granule cell excitability and the role of TRPC6 therein, using the same ramp depolarization protocol as above. In WT granule cells, bath application of hyperforin (3 µM) produces a biphasic response, where a transient increase in excitability is followed by strong inhibition of AP firing (Fig. 3A, upper traces Fig. 3C, *n* = 8 from 7 mice)., The corresponding trajectory of the membrane potential displays an initial small depolarization and a subsequent pronounced hyperpolarization which outlasts the time of drug application (Fig. 3B). By contrast, hyperforin fails to excite TRPC6-deficient neurons, while the inhibitory action of the drug remains unaffected (Fig. 3A, lower traces; Fig. 3C, *n* = 5 from 5 mice). To exclude a potential synaptic effect of hyperforin on cell excitability, we functionally isolate the recorded cells from excitatory and inhibitory inputs in WT slices with antagonists for the ionotropic glutamate receptors (kynurenic acid; 2 mM) and GABA_A_ receptors (picrotoxin,100 µM), and observe similar biphasic responses upon hyperforin application (Fig. 3C, *n* = 6 from 4 mice). During the early excitatory phase, *R*_N_ increases from 240.0 ± 10.4 MΩ under control conditions to 257.8 ± 8.6 MΩ (*p* = 0.046), and then drops to 160.5 ± 12.5 MΩ during the inhibitory phase (*n* = 6 from 4 mice, *p* = 0.002). From these findings, it becomes evident that, firstly, hyperforin requires the presence of TRPC6 to excite granule cells and promote their firing, and, secondly, that the delayed inhibitory effect of hyperforin does not depend on the preceding activation of TRPC6. When recorded in WT granule cells that were voltage-clamped at −70 mV in the presence TTX (1 µM) to block APs, hyperforin (3–10 µM) elicits an inward current of −8.0 ± 1.4 pA (*n* = 10 from 6 mice) which is followed by an outward current, while in TRPC6-deficient granule cells, the inward current response to hyperforin is abrogated (Fig. 3D, *n* = 7 from 3 mice). The hyperforin-induced outward current that is equally present in WT and TRPC6 KO neurons is probably mediated by K^+^ channels, as it is strongly reduced when we substitute Cs^+^ for K^+^ in the pipette solution (*n* = 10 from 4 wt mice; *n* = 6 from 3 TRPC6 KO mice; Fig. 3E). The exact characterization of the outward current and its possible implication in hyperforin’s antidepressant action awaits further study. However, firm conclusions can be drawn regarding the contested ionic mechanism of the hyperforin-mediated inward current. Apart from TRPC6 channels [24], anion channels [37] and proton channels [8] have been proposed to give rise to this current. Our data now strongly argue in favor of TRPC6. Moreover, they suggest that selective pharmacological activation of TRPC6 might be a promising approach to correct the aberrant firing pattern of hippocampal neurons associated with depression-like behavior.

### The C-terminal LLKL motif in TRPC6 is important for hyperforin interaction

**Fig. 4:**
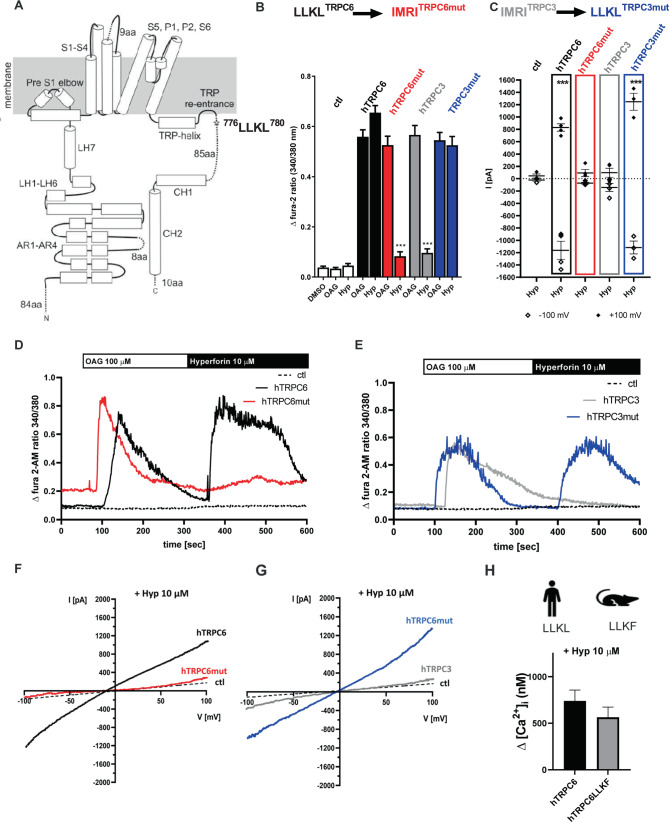
Identification of the hyperforin binding site at TRPC6 channels. **A** The topology model of human TRPC6 (hTRPC6) shows α-helices in cylinders and dashed lines describe region with not sufficient density in CryoEM structure PDB: 6uz8. Potential hyperforin bindings site is marked with a red star. **B** Sketch demonstrating that amino acids LLKL were mutated in hTRPC6 into the respective amino acids IMRI of hTRPC3 to block hyperforin-mediated TRPC6 activation. In a second step, the amino acids IMRI in hTRPC3 were mutated into the corresponding amino acids LLKL of hTRPC3 to induce a hyperforin-sensitive hTRPC3 channel. hTRPC6 (black), TRPC6mut = IMRI^TRPC6mut^ (red), hTRPC3 (gray), TRPC3mut = LLKL^TRPC3mut^. **C** Single-cell Ca^2+^ imaging was conducted in HEK293 cells transiently expressing pcDNA3.1 plasmid vector with DNA coding only for eYFP (ctl, white), hTRPC6 (black), hTRPC6mut (red), hTRPC3 (gray), or hTRPC3mut (blue) all expressed as C-terminal eYFP fusion proteins. Cells were stimulated with the solvent DMSO (0.1%), OAG (100 µM) or hyperforin (10 µM) and intracellular Ca^2+^ alterations were detected using fura-2 AM (*n* = 7–9 ± SEM, cells were selected according to their eYFP fluorescence and their OAG sensitivity; Statistical significance was analyzed by ANOVA with post hoc Dunnett’s test ***p < 0.001) **C** Whole-cell currents were recorded from HEK293 cells transiently expressing eYFP (ctl, white), hTRPC6 (black), hTRPC6mut (red), hTRPC3 (gray), or hTRPC3mut (blue) all expressed as C-terminal eYFP fusion proteins. Mean current density are depicted at +100 and −100 mV after application of hyperforin (10 µM). Currents were normalized to the basic currents before compound application were subtracted (*n* = 3 ± SEM¸ Statistical significance was analyzed by ANOVA with post hoc Dunnett’s test ***p < 0.001). **D** Representative time traces were monitored in HEK293 ctl cells (dashed line), hTRPC6-expressing HEK293 cells (black) or hTRPC6mut (red) stimulated with OAG (100 µM) 60 s after starting the experiment and after 300 s hyperforin (10 µM) was applied. **E** Representative time traces were monitored in HEK293 ctl cells (dashed line), hTRPC3-expressing HEK293 cells (gray) or hTRPC3mut (blue) stimulated with OAG (100 µM) 60 s after starting the experiment and after 300 s hyperforin (10 µM) was applied. **F** Whole-cell currents recorded from HEK293 ctl cells (dashed line), hTRPC6-expressing HEK293 cells (black) or hTRPC6mut (red). Application of hyperforin (10 µM) resulted in an increase in outward and inward current in hTRPC6 expressing cells. This effect is lost in TRPC6mut expressing cells. **G** Whole-cell currents recorded from HEK293 ctl cells (dashed line), hTRPC3-expressing HEK293 cells (gray) or hTRPC3mut (blue). Application of hyperforin (10 µM) showed no effect in ctl and hTRPC3 expressing cells but resulted in an increase in outward and inward current in hTRPC3mut expressing cells. **H** The hyperforin binding site LLKL at human hTRPC6 differs in the last amino acid from rat and mouse TRPC6 LLKF. To test if this amino acid interferes with hyperforin binding to TRPC6, we compared hTRPC6 with hTRPC6 LLKF. Single-cell calcium imaging was conducted in HEK239 cells transiently expressing hTRPC6 or hTRPC6 LLKF. Cells were stimulated with hyperforin (10 µM) and Fura-2-AM 340/380 nm ratio changes were analyzed and afterward converted into intracellular Ca^2+^ in nM. No significant differences were observed (*n* = 3 ± SEM, cells were selected according to their eYFP fluorescence; statistical significance was calculated using unpaired *t*-test, not significant 0.0576).

After demonstrating that the hyperforin-initiated inward current is lost in TRPC6 KO mice, we ask whether hyperforin directly binds to TRPC6. We previously demonstrated that hyperforin only activates human TRPC6 (hTRPC6) channels (topology model, Fig. 4A) but shows no stimulatory effect on the phylogenetically closely related hTRPC3 and hTRPC7 channels in the same subgroup or indeed any other hTRPC channels [9]. To exploit this for the identification of amino acids relevant to hyperforin-dependent TRPC6 activation, we mutate residues that differ between hTRPC6 and hTRPC3/TRPC7 (Supplemental 1, sequence alignment) [3]. These mutant hTRPC6 proteins are expressed in HEK293 cells as C-terminal YFP fusion proteins to control for expression and cellular localization. Protein expression is checked using western blot analysis and fluorescence microscopy demonstrating that no differences are found between hTRPC6 and mutated TRPC6 (Supplemental Fig. 2). To test functionality, single cell calcium measurements using the fluorescence dye fura-2 are conducted by applying OAG or SAG, analogs of the endogenous unselective TRPC3/6/7 activator diacylglycerol [38]. For calcium imaging experiments we use OAG (100 µM) and apply it prior to the application of hyperforin (10 µM). In TRPC6‐expressing cells, application of OAG provokes a rapid increase in [Ca^2+^]_i_ reflected by an increase of the fura-2 ratio. The subsequent application of hyperforin results in a response with comparable increase in [Ca^2+^]_i_. To see whether the TRPC6 mutants react differently to hyperforin, they are also stimulated with OAG (100 µM) and hyperforin (10 µM) (see Supplemental 1 alignment with all investigated mutants). Importantly, we identify a TRPC6 mutant that still showed OAG (100 µM) induced rapid increase in fura-2 ratio but does not react to hyperforin (10 µM) (Fig. 4B, D). Here, amino acids ^777^LLKL^780^ in the C-terminal region of hTRPC6 are replaced by the corresponding amino acids ^708^IMRI^711^ of hTRPC3 and hTRPC7 (^722^IMRI^725^) (topology model, Fig. 4A).

To further characterize the LLKL motif in the TRPC6 C-terminus as a putative binding site of hyperforin, we test the hTRPC6 mutant in electrophysiological recordings. Whole‐cell recordings of hTRPC6‐expressing HEK293 cells result in current‐voltage relationships comparable with earlier data (Fig. 4C, F). In standard extracellular solutions, the current‐voltage relationship of the SAG current (Supplemental Figs. 3A, B, and 2C), measured from voltage ramps, has an outwardly rectifying form, comparable with the curves resulting from hyperforin application (Fig. 4F). Hyperforin exhibits no effect on untransfected HEK293 cells (Fig. 4F). The hTRPC6 ^708^IMRI^711^ mutant still presents the properties of the current‐voltage relationship of the SAG current (Supplemental Fig. 3B, C) but does not react to hyperforin (10 µM) (Fig. 4C, F).

Since TRPC6 and TRPC3 share structural and sequential homology, we wondered whether TRPC3, which normally does not respond to hyperforin, can be sensitized to this compound by transplanting the LLKL motif from TRPC6 into TRPC3. As expected, WT hTRPC3 can neither be activated by hyperforin in single-cell calcium imaging experiments nor in whole-cell patch-clamp recordings. The addition of OAG (100 µM) leads to a robust Ca^2+^ influx in HEK cells transiently expressing hTRPC3 (Fig. 4B, G). In contrast, hyperforin induces an inward current in the hTRPC3 mutant carrying the LLKL binding motif (Fig. 4C, G) showing that this motif is necessary and sufficient to endow TRPC3 or TRPC6 with hyperforin sensitivity.

Since our behavioral experiments were carried out in mice, we further investigate hyperforin-mediated activation of murine TRPC6 shown in Fig. 4H to complement the cell-based data on human hTRPC6 described above. In patch clamp and in single-cell calcium imaging experiments, we previously obtained no obvious differences between the hyperforin-mediated outward currents or Ca^2+^ influx in the two species. However, the last amino acid of the human TRPC6 hyperforin binding motif LLKL is altered to LLKF in murine TRPC6 (see Supplemental Figs. 1 and 4H). We thus check whether a leucine to phenylalanine substitution in the human TRPC6 channel affects hyperforin activation. Using HEK293 cells transiently expressing the hTRPC6 LLKF mutant, we only observe minor changes in single-cell calcium imaging experiments compared to WT hTRPC6 (Fig. 4H). These results suggest that the last amino acid of the LLKL binding motif might only play a minor role in hyperforin-mediated hTRPC6 activation.

**Fig. 5:**
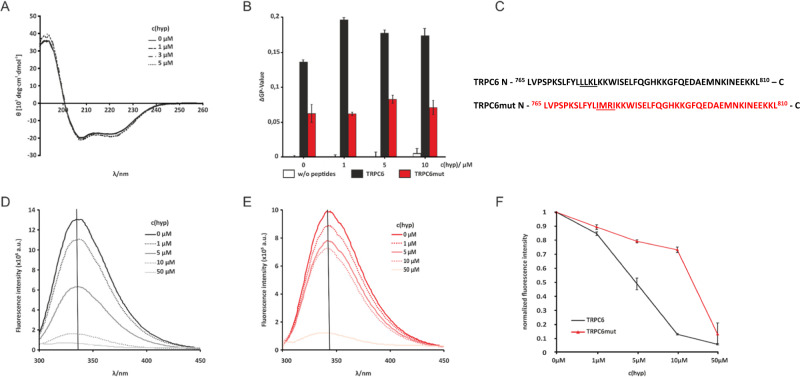
Interaction studies of TRPC6 C-terminal peptides with hyperforin and membranes. CD spectra of wild-type TRPC6 peptides in the absence and presence of hyperforin (**A**). Laurdan fluorescence measurement to monitor membrane fluidity changes caused by hyperforin (white bars), TRPC6 peptide (black bars), and TRPC6mut peptide (red bars) (**B**). Amino acid sequences from TRPC6 and TRPC6mut are shown in (**C**). Differences between the two sequences are underlined. Tryptophan fluorescence using residue W782 as a reporter of TRPC6 (**D**) and TRPC6mut titrated with hyperforin (**E**). Fluorescence maxima (vertical black line in **D** and **E**) were blotted against hyperforin concentration and normalized against fluorescence maxima without hyperforin (**F**).

The binding site of hyperforin within the hTRPC6 C-terminus, the ^777^LLKL^780^ motif, is localized intracellularly in a region of the channel (residues 768–853) not resolved in the reported cryo-EM structures of hTRPC6 (Fig. 4 topology model) [19, 20]. Therefore, it is suspected that these structures are disordered or highly flexible. To identify the overall secondary structure of TRPC6 C-terminal peptide and to examine potential structural changes upon hyperforin interaction, we measured CD spectra of isolated peptides carrying the ^777^LLKL^780^ motif or its mutated ^777^IMRI^780^ stretch (Fig. 5A, C). The TRPC6 peptide is α-helical structure in the absence and presence of hyperforin (5 µM) (Fig. 5A), but no changes in the overall secondary structure in response to increasing hyperforin concentrations are observed. Due to interference of hyperforin with circulated light, addition of hyperforin to concentrations higher than 5 µM is not possible. This TRPC6 C-terminal peptide contains numerous hydrophobic residues, which might indicate that it can interact with lipids. Thus, we monitor interaction of the TRPC6 C-terminus and its mutant with lipid membranes, the influence of hyperforin on this interaction, and whether hyperforin directly interacts with the isolated TRPC6 C-terminal region (Fig. 5) by using either the membrane-permeable fluorescent dye Laurdan (Fig. 5B), which monitors water accessibility and thus acts as a proxy for membrane fluidity [39] or the native tryptophan residue W783 of the peptides for fluorescence measurements (Fig. 5D–F).

Laurdan fluorescence measurement is a tool to monitor fluidity changes in liposomes or membranes. Generalized polarization (GP) values of POPC-POPG-liposomes with Laurdan were recorded upon addition of hyperforin and in the presence of two peptides representing a native TRPC6 C-terminal region harboring the LLKL motif or the corresponding residues ^708^IMRI^711^ from TRPC3 (Fig. 5C, TRPC6mut). In the presence of up to 10 µM hyperforin ΔGP values of the POPC-POPG liposomes do not change (Fig. 5B, white bars). Thus, hyperforin does not influence membrane fluidity on its own under these conditions. In contrast, addition of either TRPC6 peptide results in a strong increase in the respective ΔGP values, indicating a rigidification of the membrane caused by peptide binding. Interestingly, the effect of the mutated TRPC6 peptide is less pronounced than that of the WT peptide (Fig. 5B, red vs. black bars). Upon addition of hyperforin to the liposome peptide complex, the ΔGP value with the WT TRPC6 peptide increases even more, while the ΔGP values with TRPC6mut remain stable. Thus, WT TRPC6 peptide and hyperforin synergistically lead to reduced membrane fluidity already at low hyperforin concentrations. To investigate whether hyperforin can directly interact with the peptides, we use tryptophan fluorescence by taking advantage of the only native tryptophan residue, W783, which is located next to the proposed hyperforin binding site. Tryptophan fluorescence is very sensitive to changes in the environment such as hydrophobicity and can thus report on ligand binding. Upon titrating hyperforin to the two TRPC6 peptides, a decrease in fluorescence intensity is observed (Fig. 5D–F). Importantly, the TRPC6mut peptide shows a significantly reduced interaction with hyperforin thus indicating that the LLKL motif indeed plays an important role in hyperforin interaction.

### Hyp13 activates TRPC6 channels

**Fig. 6:**
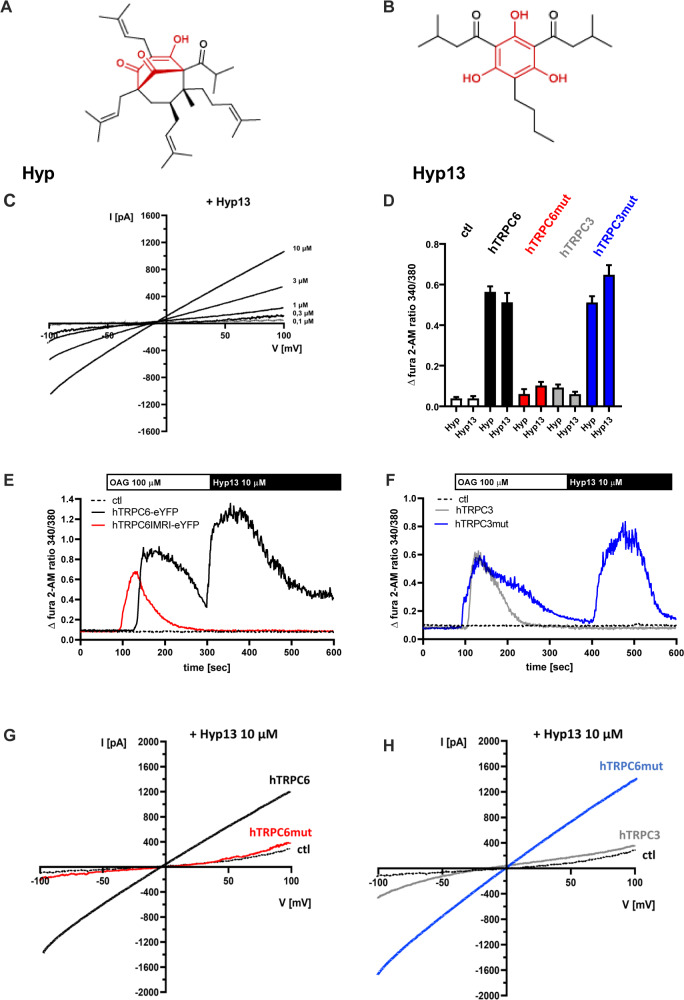
Characterization of the simplified hyperforin-derivative Hyp13. Chemical structure of hyperforin (**A**) and Hyp13 (**B**). The phloroglucinol core structure is highlighted in red. (**C**) Concentration dependent effect of Hyp13 in HEK293 cells expressing hTRPC6 channels in whole cell patch clamp experminents. **D** Hyp13 also interacts to the LLKL binding motif at TRPC6. Single-cell Ca^2+^ imaging was conducted in HEK293 cells transiently expressing pcDNA3.1 plasmid vector with DNA coding only for eYFP (ctl, white), hTRPC6 (black), hTRPC6mut (red), hTRPC3 (gray), or hTRPC3mut (blue) all expressed as C-terminal eYFP fusion proteins. Cells were stimulated with the hyperforin (10 µM) or Hyp13 (10 µM) and intracellular Ca^2+^ alterations were detected using fura-2 AM (*n* = 7–9 ± SEM, cells were selected according to their eYFP fluorescence and their OAG sensitivity). **E** Representative time traces were monitored in HEK293 ctl cells (dashed line), hTRPC6-expressing HEK293 cells (black) or hTRPC6mut (red) stimulated with OAG (100 µM) 60 s after starting the experiment and after 300 s hyperforin (10 µM) was applied. **F** Representative time traces were monitored in HEK293 ctl cells (dashed line), hTRPC3-expressing HEK293 cells (gray) or hTRPC3mut (blue) stimulated with OAG (100 µM) 60 s after starting the experiment and after 300 s hyperforin (10 µM) was applied. **G** Whole-cell currents recorded from HEK293 ctl cells (dashed line), hTRPC6-expressing HEK293 cells (black) or hTRPC6mut (red). Application of Hyp13 (10 µM) resulted in an increase in outward and inward current in hTRPC6 expressing cells. This effect is lost in TRPC6mut expressing cells. **H** Whole-cell currents recorded from HEK293 ctl cells (dashed line), hTRPC3-expressing HEK293 cells (gray) or hTRPC3mut (blue). Application of Hyp13 (10 µM) showed no effect in ctl and hTRPC3 expressing cells but resulted in an increase in outward and inward current in hTRPC3mut expressing cells.

Hyperforin (Fig. 6A) is a polyprenylated bicyclic acylphloroglucinol derivative that is not very stable when exposed to light and oxygen and induces drug-drug interactions [30]. Therefore, we previously prepared simplified 2,4-diacylphloroglucinols which are chemically stable and demonstrate similar activity and selectivity for TRPC6 but do not activate *PXR* [30]. Here, we characterize the effects of a novel phloroglucinol derivative, Hyp13 (Fig. 6B), on TRPC6-mediated inward currents in HEK293 cells transiently expressing TRPC6 channels. The current-voltage relationship of the Hyp13-induced current, measured from voltage ramps, has an outwardly rectifying form, comparable with the curves resulting from hyperforin application (Fig. 6C). Hyp13 (10 µM) is selective for TRPC6 (Fig. 6D) and does not activate TRPC3 (Fig. 6E). In HEK293 cells transiently expressing hTRPC6, Hyp13 (10 µM) induces in single cell calcium experiments a robust and transient Ca^2+^ influx (Fig. 6D, E) comparable to the effects of hyperforin. In hTRPC3 expressing HEK293 cells, Hyp13 (10 µM) similar to hyperforin (10 µM) does not elicit Ca^2+^ influx in single cell calcium imaging experiments (Fig. 6D, F). In whole-cell patch-clamp experiments in hTRPC6 expressing HEK293 cells, Hyp13 (10 µM) initiates an outward rectifying current (Fig. 6G) comparable to the effects of hyperforin (10 µM). Hyp13 (10 µM) shows no effects on hTRPC3 expressing HEK293 cells (Fig. 6D, H). In addition, the Hyp13 induced Ca^2+^ influx (Fig. 6D, E) and outward rectifying channel is lost in HEK293 cells transiently expressing the TRPC6 IMRI mutant (Fig. 6G). In contrast, in HEK293 cells expressing the TRPC3 LLKL mutant the Ca^2+^ influx (Fig. 6D, F) and outward rectifying channel induced by Hyp13 is restored (Fig. 6H). These results suggest that hyperforin and Hyp13 share the same binding site in hTRPC6.

### No induction of CYP3A4 expression by Hyp13

**Fig. 7:**
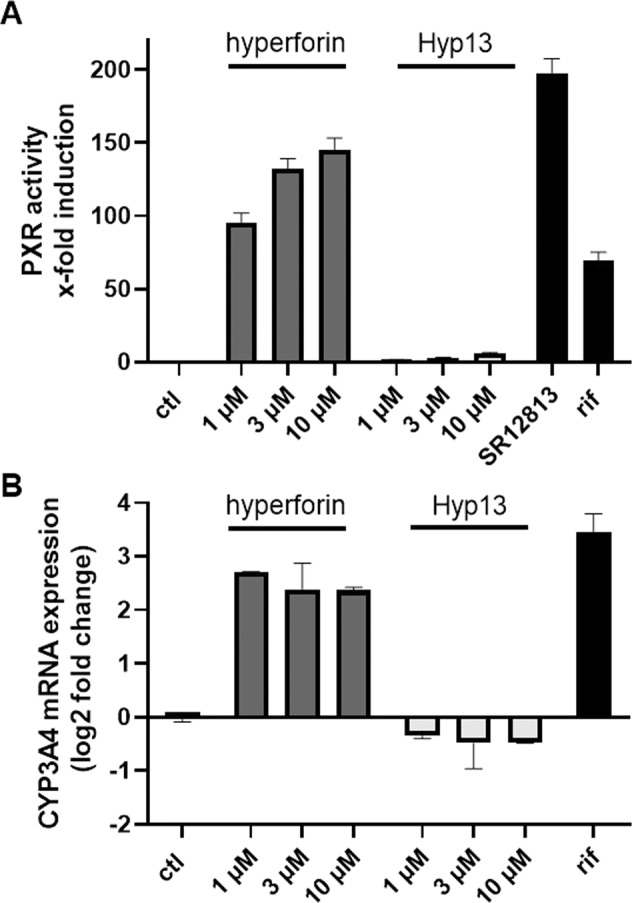
Hyp13 does not activate *PXR*. Induction of *PXR* activity by hyperforin and its derivate in HepG2 cells (**A**). HepG2 cells were co-transfected with plasmids expressing GAL4-responsive UAS-driven firefly luciferase, human *PXR*-LBD fused to GAL4-DBD, and Renilla luciferase. The transfected cells were exposed to 10 µM of positive controls rifampicin and SR12813 or different concentrations of hyperforin and its derivate. After 24 h, cell lysates were assayed for firefly and Renilla luciferase activity. Firefly luciferase activity was normalized against Renilla luciferase activity and fold induction relative to the solvent control (SC 0.5% DMSO) was calculated. Data are presented as means ± SD of three independent experiments performed with six replicates each. **B** Gene expression analysis of *CYP3A4*. Differentiated HepaRG cells were exposed to hyperforin and its derivate as well 10 µM Rifampicin (PC) for 24 h. Total mRNA was isolated and transcribed into cDNA and subsequently mRNA expression of *CYP3A4* was analyzed by real-time qPCR. For relative quantification, Ct values were normalized to reference genes (*ACTB* and *GAPDH*) according to the ΔΔCT method. Log2 fold changes of 2^−ΔΔCT^ values were calculated and mRNA levels were expressed in relation to the solvent control (SC 0.5% DMSO). Data are presented as means ± SD of two to three independent experiments.

To test if the drug-drug interaction properties of hyperforin are also lost in our newly characterized phlorogucinol derivative, we investigated if Hyp13 activates *PXR* using a GAL4 fusion construct-based assay aimed to monitor binding of a substance to the *PXR* ligand-binding domain [40, 41]. Before starting this experiment, we investigated the cytotoxicity of Hyp13 compared to hyperforin after 24 h treatment (Supplemental Fig. 4). Hyp13 induced cytotoxic effects comparable to hyperforin in HepG2 and HepaRG cells. As described previously, hyperforin strongly and concentration dependently activates *PXR* such as the well-known *PXR*-agonists SR12813 and rifampicin (Fig. 7A, Supplemental Fig. 4). In contrast, nearly no activation was observed after treatment with Hyp13 (Fig. 7A, Supplemental Fig. 4).

Subsequently, *CYP3A4* mRNA expression was analyzed (see Fig. 7B) and revealed a strong activation of *CYP3A4* by hyperforin in a level comparable with that of the positive control rifampicin, whereas no pronounced effect was caused by Hyp13.

### TRPC6 channels are essential for the antidepressant effect of Hyp13

**Fig. 8:**
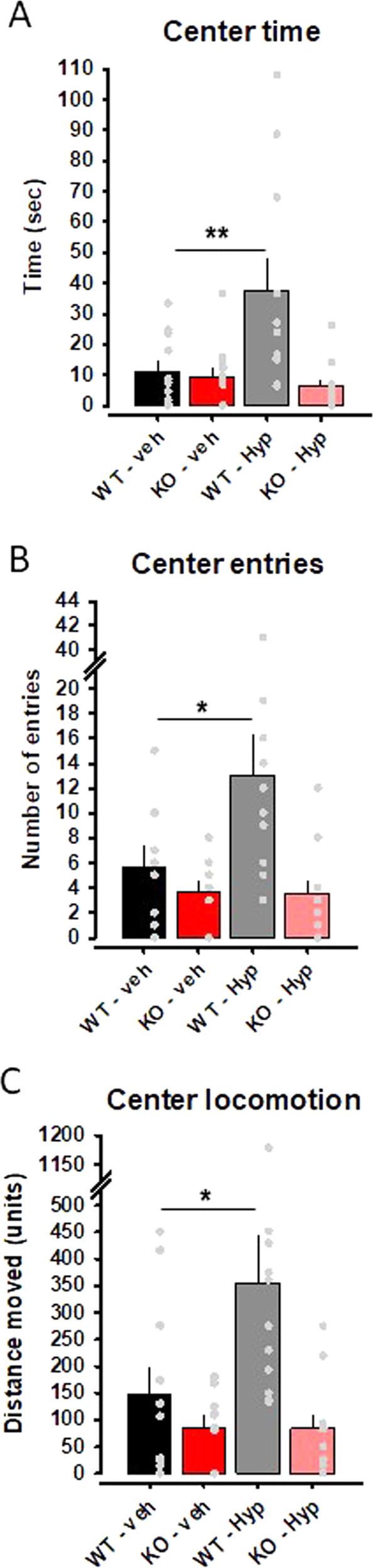
TRPC6 channels are essential for Hyp13-mediated anxiolytic effects. Anxiolytic effects of Hyp13 (Hyp) are TRPC6 dependent. Anxiolytic effects were shown in the open field test as measured by the **A** center time, **B** center entries, and **C** center locomotion. TRPC6 knock out (KO) mice do not show these effects. Data are expressed as means ± s.e.m. (*n* = 11–12 per group; *p < 0.05, **P < 0.001).

Most importantly, we test whether TRPC6 channels are essential for Hyp13-mediated anxiolytic and antidepressant effects. Hyp13 shows antidepressant and anxiolytic effects in male B6J;129S8 WT mice in several tests indicative for depression and anxiety, such as the open field test (Supplementary Fig. 5), novelty suppressed feeding (Supplementary Fig. 6), or forced swim test (Supplementary Fig. 7). We focus on one behavioral paradigm which was strongly affected in male TRPC6 KO mice, the open field test. We find significantly increased center time, center locomotion, and center entries at a concentration of 5 mg/kg in WT mice. With these results, we apply Hyp13 (5 mg/kg) to TRPC6 KO mice and observe a loss of the anxiolytic effects of Hyp13 in the open field test compared to WT mice (Fig. 8). These findings suggest that the emotional effects of Hyp13 are TRPC6-dependent.

## Discussion

In this study, we characterize the role of the TRPC6 channel for the antidepressant effects of hyperforin. We report that TRPC6 KO mice show an enhanced depression- and anxiety-like behavior. One likely mechanism for this phenotype is the observed change in the excitability in DG and CA1 neurons of the hippocampus. We demonstrate that hyperforin excites hippocampal neurons in a TRPC6-dependent fashion. Subsequently we identified a binding motif essential for hyperforin in the TRPC6 C-Terminus. To overcome the disadvantageous chemical properties of hyperforin as an antidepressant lead compound, we synthetized the phloroglucinol derivative Hyp13. We show that the hyperforin analog Hyp13 acts through the hyperforin binding motif on TRPC6. Finally, we provide evidence that Hyp13 has antidepressant actions depending on TRPC6 channels.

TRPC6 KO mice showed enhanced anxiety-like responses in the open field and elevated plus maze test and displayed increased depression-like behavior in the forced swim test and in the sucrose preference test reflecting the animal’s capacity to experience hedonic pleasure. Until now, depression-related behavior was not investigated in TRPC6 KO mice. However, chronic unpredictable stress combined with isolation in rats lead to reduced TRPC6 expression in the hippocampus [42]. Diminished synaptic plasticity was reflected by an impaired LTP, reduction in dendritic length and density of spines, as well as by lowered postsynaptic density 95 (PSD-95) staining, a surrogate for the density of post-synapses. Recently published data suggest that viral knockdown of TRPC6 in the DG of mice has a disadvantageous effect on cognitive processes and anxiety-like behavior [43]. Similarly, to our results, shRNA-TRPC6 treated animals spent less time in the central area of the open field test than the control group indicating anxiety-like behavior. The mice in the shRNA-TRPC6 group were also unable to discriminate between novel and familiar mice in the three-chamber test and exhibited impaired spatial learning and working memory. Others have described the impact of TRPC6 for learning and memory. Using the square open field and the elevated star maze test, reduced exploratory activity was found in TRPC6 KO mice [44, 45]. In the Morris Water Maze, transgenic mice overexpressing TRPC6 showed enhancement in spatial learning and memory [26]. Keeping in mind that altered cognition is one of the symptoms of major depression and major depression is one of the main risk factors for dementia, TRPC6 might be a link between these psychiatric diseases. Several studies demonstrated that the selective TRPC6 activator hyperforin reduces Aβ levels and improves behavioral performance in Alzheimer’s disease (AD) animal models and in vitro studies [46]. Moreover, reduction of TRPC6 expression was found in patients with AD and mild cognitive impairment, which negatively correlated with the cognitive performance [10].

The observed anxious and depression-like behavior of TRPC6 KO mice was accompanied by reduced neuronal excitability in DG granule cells and CA1 pyramidal neurons. This altered excitability coincides with TRPC6 channel expression which are mainly expressed in DG neurons, pyramidal neurons, and interneurons of the hippocampus. The hippocampus is a highly stress-sensitive brain region and its dysfunction may contribute to the deficits in concentration among the diagnostic features of major depression [47]. The response to novelty is regulated by hippocampal afferents projecting to the nucleus accumbens and the ventral tegmental area [48]. Impaired hippocampal function is also discussed to be involved in anhedonia [49]. DG cells and CA1 pyramidal cells from TRPC6 KO mice demonstrate a significantly reduced cell excitability. Similar data were obtained by Griesi-Oliveira *et al*. downregulating TRPC6 in mouse primary neurons using shRNA [45]. However, since TRPC6 channels are mainly expressed in interneurons in CA1 [23], the reduced excitability in CA1 neurons in TRPC6 KO mice might be also be induced by a homeostatic mechanism due to chronic lack of TRPC6 in CA1 interneurons. Whole-cell recordings of APs revealed a significant reduction in the firing rate of neurons expressing shRNAs against TRPC6 compared to controls. Notably, aberrant AP firing in response to membrane depolarization has also been observed in iPSC-derived DG-like neurons from patients with bipolar disorder [50]. Airan et al. provided evidence that DG neurons show dynamic changes in models of depression and after chronic SSRI treatment [51]. Using voltage-sensitive dye imaging, DG activity was reduced in a chronic mild stress model and was reversed after chronic fluoxetine treatment. This reduced activity is probably mediated by atrophy in the dendrites of DG. Several groups demonstrated a role of TRPC6 for dendritic spine morphology and arborization. Using adeno-associated viral vectors (AAV) expressing short hairpin RNA (shRNA) targeting TRPC6 in the hippocampal DG of male mice, reduced TRPC6 protein expression and diminished dendritic spines of DG granule neurons [43]. These data suggest that the anxious and depressive-like behavior in TRPC6 KO mice might be caused by reduced excitability in DG neurons associated with a reduced number of spines and dendrites. In contrast, hyperforin improves synaptic plasticity in the hippocampus and prefrontal cortex by altering spine morphology [24] and the expression of CREB [52, 53], BDNF [54], and TrKB receptors [42, 55].

While previous studies, comprising the FST, learned helplessness paradigms, avoidance deficit models, and the EPM demonstrated antidepressant and anxiolytic effect of hyperforin [42, 54, 56–58], a recent report questioned whether hyperforin is a specific TRPC6 activator [7, 8]. In this study, hyperforin increased the excitability in the DG neurons in WT mice. This effect was abrogated in TRPC6 KO mice, as was the hyperforin-induced inward current. Since the hyperforin-induced delayed potassium-mediated current persists in TRPC6 KO mice, this might be an independent mechanism of action. To further investigate the interaction of hyperforin with TRPC6, we performed site-directed mutagenesis experiments focusing of amino acids which differ between TRPC6 and the closely related TRPC3 and TRPC7 channels that cannot be activated by hyperforin. Thus, we were able to identify a binding motif in the TRPC6 C-terminus, ^777^LLKL^780^, that mediates hyperforin sensitivity. These four residues, which are not conserved within the TRPC family, are located between the TRP re-entrant and C-terminal helix 1, a region with disordered amino acids (766–854) in cryo-EM structures [19, 22]. This region also contains the putative inositol hexaphosphate (842–868) and the calmodulin/IP3 receptor (838–872) binding sites [59]. The increase of TRPC6-mediated Ca^2+^ influx by SAG and OAG, both analogs of the native lipid agonist DAG, was not altered in the mutant channels. Our results suggest that neither protein folding, trafficking or surface expression of TRPC6 is affected by the hyperforin binding motif ^777^LLKL^780^.

Furthermore, we show that upon isolation, this region is α-helical and interacts with liposomes and affects membrane fluidity. Tryptophan fluorescence spectroscopy taking advantage of a native residue in the vicinity of the proposed hyperforin binding site indicates that there is indeed a direct interaction between TRPC6 C-terminal region and hyperforin. This interaction is reduced when the ^777^LLKL^780^ motif is mutated. Interestingly, the membrane interaction and resulting fluidity changes are also enhanced by the presence of hyperforin.

Since hyperforin shows several shortcomings such as poor chemical stability or the induction of drug-drug interactions as a lead compound for further developments [29], we synthetized and investigated a chemically simplified and stable hyperforin analog, which shares the phloroglucinol core, Hyp13. Hyp13 is based on the previously published Hyp derivatives from our group and shares all features with hyperforin. We showed that Hyp13 activates TRPC6 in similar potency, shows no effect on TRPC3, and binds to the identified binding motif LLKL. Furthermore, Hyp13 in contrast to hyperforin does neither activate *PXR* nor induced *CYP3A4*. These findings indicate that Hyp13 has a favorable interaction profile compared to hyperforin which is known to lead to serious drug-drug interactions. However, hyperforin and Hyp13 showed similar cytotoxic effects at concentrations higher than 5 µM after 24 h in HepG2 and HepaRG cells. These concentrations are far above the concentrations reached in behavioral experiments in mice. For hyperforin, nanomolar brain concentrations where observed after oral treatment in mice matching with concentrations needed to alter synaptic plasticity via the activation of TRPC6 channels [24, 60]. Importantly, Hyp13 exhibits antidepressant- and anxiolytic-like effects in the open field test, the novelty suppressed feeding test, and the forced swim test. It should be noted that these effects were observed already after acute treatment, while hyperforin in humans requires several weeks to display full antidepressant action. This might suggest a faster action of Hyp13 compared to hyperforin. However, the applied tests show a predictive validity in that they indicate drug action, but not on the human time scale, as shown for other antidepressant drugs [61]. The emotional effects of Hyp13 were reduced in TRPC6 KO mice.

## Conclusions

In summary, we demonstrate the role of TRPC6 channels for depression and anxiety and provide evidence for TRPC6 channels as novel druggable targets for therapies in mood disorders. These results might also pave the way for new molecules to treat other psychiatric diseases associated with TRPC6 dysfunction such as autism or Alzheimer disease.

## Methods

### Sources and preparation of reagents

Hyperforin was kindly supplied by Dr. Willmar Schwabe (Karlsruhe, Germany). Hyperforin and Hyp13 were diluted in dimethyl sulfoxide (DMSO; Carl Roth, Karlsruhe, Germany #A994.1) stored until use at –20 °C. 1-oleoyl-2-acetyl-sn-glycerol (OAG; Sigma-Aldrich, Taufkirchen, Germany; #O6754) was solved in DMSO and stored at 100 mM stock solution, 1-stearoyl-2-arachidonoyl-sn-glycerol-d8 (SAG; Santa Cruz Biotechnology Inc., Santa Cruz, CA, USA; #sc-220503) was solved in DMSO and stored at 50 mM stock solution at –20 °C. Rifampicin (CAS 13292-46-1) and SR12813 (CAS 126411-39-0) were purchased from Sigma-Aldrich (Taufkirchen, Germany). Standard laboratory chemicals were obtained from Sigma-Aldrich or Carl Roth.

### Synthesis and characterization of Hyp13

Hyp13 was synthesized according to Bharate et al. [62]. was realized in a two-step sequence starting from phloroglucinol, which was first treated with isovaleryl chloride under Friedel-Crafts conditions to afford Hyp1. Subsequently, Hyp13 was obtained by alkylation of Hyp1 with 1-iodobutane in the presence of sodium methoxide as a base and chromatographic separation of the formed isomers.

### Site-directed mutagenesis

pcDNA3.1 plasmid vector with DNA coding for human TRPC6 (Uniprot ID: Q9Y210) and human TRPC3 (Uniprot ID: Q13507), both C-terminally fused to eYFPs, were used as the templates to hTRPC6 ^777^IMRI^780^-eYFP and hTRPC3 ^708^LLKL^711^-eYFP mutants. For site-directed mutagenesis, we used primer sequences (custom made by Sigma Aldrich, Germany) coding for the planned mutations. Primer sequences were as follows:

### TRPC6 777IMRI780 forward primer:

5′TCTGGTGCCGAGTCCAAAGTCCCTGTTTTATCTCATAATGCGCATTAAAAAATGGATTTCTGAGCTGTTCCAGGGCC

### TRPC6 777IMRI780 reverse primer:

5′GCTCAGAAATCCATTTTTTAATGCGCATTATGAGATAAAACAGGGACTTTGGACTCGGCACCAGATTGAAGGGTACAGG;

### TRPC3 708LLKL711 forward primer:

5′-GTTTATTTCCTCCTGAAACTTGTTAACTTTCCCAAATGCAGAAGGAGAAG;

### TRPC3 708LLKL711 reverse primer:

5′-TGGGAAAGTTAACAAGTTTCAGGAGGAAATAAACAAATGATTTTGGACTAGG.

PCRs were carried out using the following conditions: initial denaturation for 3 min at 95 °C and 25 cycles of denaturation for 20 s at 98 °C, annealing between 75–80 °C for 15 s and extension at 72 °C for 5 min. Amplification was performed using high-fidelity DNA Polymerase (Roche, Mannheim, Germany; #KK2101). Template DNA was removed by digestion using DpnI (New England Biolabs, Frankfurt am Main, Germany; # R0176S) and amplified DNA was used for the transformation of competent DH5α *Escherichia coli cells* (ThermoFisherScientific, Darmstadt, Germany; #18265017). Plasmid DNAs from several clones were isolated from pre-poured agar plates with 100 μg/mL ampicillin and confirmed by Sanger sequencing. For transfection, large–scale DNA preparations were performed using Plasmid Maxiprep Kit (Zymo Research Europe GmbH, Freiburg im Breisgau, Germany; #D4202).

### Cell culture and transfection

Human embryonic kidney (HEK 293) cells were cultured in Dulbecco’s Modified Eagle Medium (DMEM; ThermoFisherScientific, Darmstadt, Germany; #41965039) supplemented with 10% heat-inactivated Fetal Bovine Serum (FCS; ThermoFisherScientific, Darmstadt, Germany; #10500-064) and 10 mM penicillin/streptomycin (Pen-Strep; ThermoFisherScientific, Darmstadt, Germany; #15140122) at 37 °C. For Transfection, HEK 293 cells were grown on poly-L-lysine (Sigma-Aldrich, Taufkirchen, Germany; #P2636) coated glass coverslips in 6-well plates with a density of 0.1 × 10^6^ cells per well for single-cell calcium imaging and western blot and with a density of 0.5 × 10^5^ cells for electrophysiological measurements. After 24 h, media was exchanged and cells were transfected transiently with a transfection cocktail containing 0.5–1 μg DNA, 2 μL Effectene (Qiagen, Hilden, Germany; #301425) transfection reagent, and 50 μL Opti-MEM (ThermoFisherScientific, Darmstadt, Germany; #51985034) medium. The Single-Cell Calcium Imaging, Western Blot, and electrophysiological study were carried out 24 h after transfection. All cell lines were tested negative for mycoplasma contamination using PCR test.

### Protein extract preparation and Western Blot analysis of TRPC channel expression in HEK293 cells

Transfected and untransfected HEK 293 cells were washed in phosphate-buffered saline (PBS; pH 7.3), pelleted, and lysed in Radioimmunoprecipitation assay (RIPA) buffer (50 mM Tris-HCl, 1% Triton X-100, 150 mM NaCl, 0.5% Na-deoxycholate, 0.1% sodium dodecyl sulfate (SDS), 5 mM ethylenediaminetetraacetic acid (EDTA), 1 mM phenylmethylsulfonylfluoride (PMSF) containing cCOMPLETE^TM^ protease inhibitor cocktail (Roche, Mannheim, Germany; #04693159001). Samples were lysed for 30 min at 4 °C and centrifuged at 14.000 rpm for 10 min at 4 °C. The supernatants were added to 4 × SDS-Laemmli buffer (Carl Roth, Karlsruhe, Germany #K929.1) and boiled, and proteins were separated by 8% sodium dodecyl sulfate polyacrylamide gel electrophoresis (SDS-PAGE). The gels were blotted onto PVDF membranes at 80 mV over 2 h at 4 °C. PVDF membranes were blocked over 3 h with 5% bovine serum albumin (BSA; Carl Roth, Karlsruhe, Germany; #T844.3) in TBS buffer (20 mM Tris, 150 mM NaCl) with 0.1% Tween 20, incubated with antibodies specific for TRPC6 (Alomone Labs, Jerusalem, Israel; #ACC-017) diluted 1:400-fold, TRPC3 (Cell Signaling, Frankfurt am Main, Germany; #D4P5S) diluted 1:1000-fold and ß-actin (Sigma Aldrich, Taufkirchen, Germany; #A1978) diluted 1:2000. TRPC6 and TRPC3 antibodies were diluted in TBS/0.1% Tween 20 buffer with 5% BSA and b-actin antibody was diluted in TBS/0.1% Tween 20 buffer with 1% BSA. The membranes were washed three times in TBS/0.5% Tween 20 buffer, incubated for 60 min with horseradish peroxidase coupled antibodies against the primary antibodies, washed again three times in TBS/0.5% Tween 20 buffer, and developed with the electrogenerated chemiluminescence reagent (ECL; GE Healthcare Europe, Munich, Germany; #RPN2236). Western blot visualization was carried out using the iBright CL1500 Imaging System (ThermoFisherScientific, Darmstadt, Germany).

### Electrophysiology in HEK293 cells

Whole-cell patch-clamp recordings were performed in HEK 293 cells. Patch pipettes were fabricated from borosilicate glass capillaries (Science Products, Hofheim, Germany; GB150F-10P) with a P-1000 micropipette puller (Sutter Instruments, Novato, USA), The pipette resistance varied from 4–8 MΩ. Whole-cell currents were elicited by voltage ramps from –100 to +100 mV (400 ms duration) applied every 10 s from a holding potential of 0 mV. Currents through the pipette were recorded by EPC 10 USB amplifier and patchmaster software (Patchmaster, HEKA Electronics, RRID:SCR_000034, Germany), filtered at 2.9 kHz (Bessel filter), digitized at 4 kHz, and analyzed using Fitmaster software (Fitmaster, HEKA Electronics,Lambrecht, Germany; RRID:SCR_016233) and Igor Pro 8.0 (Wavemetrics, Tigard, USA). One cell was recorded per cover slip, at least three independent cell preparations were studied for each experiment. Pipettes were filled with an intracellular solution containing 130 mM CsCH_3_O_3_S, 10 mM CsCl 2 mM MgCl_2_, and 10 mM HEPES (pH 7.2 with CsOH). The standard extracellular solution contained 135 mM NaCl, 5 mM KCl, 2 mM CaCl_2_, 1 mM MgCl_2_, 10 mM glucose, and 10 mM HEPES (pH 7.4 with NaOH).

### Single-cell calcium imaging

[Ca^2+^]_i_ measurements in HEK293 cells were carried out using the fluorescence indicator fura-2-acytoxymethyl ester (Fura 2-AM; ThermoFisherScientific, Darmstadt, Germany; #F1201) combined with a monochromator-based imaging system (T.I.L.L. Photonics; FEI, Gräfeling, Germany) attached to a fluid immersion objective (LUMPLFLN 40XW/0.80 w). Cells were loaded with a cocktail composed of 2.5 μM Fura 2-AM, 0.01% pluronic-F127 (ThermoFisherScientific, Darmstadt, Germany; #P6866) for 30 min at room temperature (22–24 °C) in a standard Hank’s Balanced Salt Solution (HBSS) buffer composed of 138 mM NaCl, 6 mM KCl, 1 mM MgCl_2_, 1 mM CaCl_2_ and 10 mM HEPES ([4-(2-hydroxyethyl)-1-piperazineethanesulfonic acid]) adjusted to pH 7.4 with NaOH at room temperature. Afterward, cells were washed with HBSS and left for another 30 min at RT in HBSS. Cover slips were then mounted in a bath chamber made of plexiglas on the stage of the microscope (Olympus BX51WI, Hamburg, Germany). Ca^2+^ influx was recorded and visualized in TillVision Live Acquisition and Offline Analysis software [formerly FEI Munich GmbH (Till Photonics), now Thermo Fisher Scientific] as a ratio of 340/380 nm with a 40× objective. Ca^2+^-bound Fura2-AM is excitable at 340 nm and the unbound state of Fura2-AM at 380 nm. The ratio was calculated by analyses of emission which was detectable at 510 nm after excitation with each wavelength.

### TRPC6 peptide preparation

hTRPC6 and hTRPC6mut peptides were synthetized by peptides & elephants (Henningsdorf, Germany). Specifically, a peptide comprising residues 766–811 of the human TRPC6 C-terminus (^766^LVPSKSLFY**LLKL**KKWISELFQGHKKGFQEDAEMNKINEEKKL^811^) and the peptide carrying the mutated motif IMRI as identified in TRPC3 (^766^LVPSKSLFY**IMRI**KKWISELFQGHKKGFQEDAEMNKINEEKKL^811^) were obtained. To remove impurities, peptides were dissolved in 50 mM Tris pH8, 150 mM NaCl and dialyzed against the same buffer for several days. To remove salts, peptides were dialyzed against Milli-Q-water for another 7 days and the final peptide concentration was set to 100 µM or lyophilized and stored at −20 °C until further use.

### CD spectroscopy

CD spectra were recorded on Jasco-815 CD spectrometer at 25 °C and in a wavelength range from 190–260 nm. TRPC6 and TRPC6mut peptides were dissolved in 10 mM Tris pH 8, 30 mM NaCl with a peptide concentration of 20 µM.

### Liposome preparation

POPC and POPG lipids in chloroform were mixed in a molar ratio of 70:30. The lipid mixture with an addition of 1-[6-(dimethylamino)−2-naphthalenyl]−1-dodecanone (Laurdan) (Laurdan:Lipid 1:500 molar ratio) was dried under nitrogen flow and vacuum to obtain a lipid film. The lipid film was resuspended in 20 mM Tris pH 7.5, 150 mM NaCl to reach a total lipid concentration of 500 µM. Small unilamellar vesicles (SUV) were prepared with five freeze-thaw cycle and subsequent extrusion through a 50 nm membrane.

### Laurdan fluorescence measurement

50 µM of the lipid/Laurdan mixture (see above) were preincubated with 5 µM TRPC6 peptides. Hyperforin with different concentration was added freshly to the mixture and the spectra were recorded using a Fluoromax-4 (Horiba Scientific) fluorimeter. Emission spectra were recorded from 400–550 nm, with an excitation wavelength of 340 nm and slit width of 1 nm at 25 °C. Fluorescence intensities at 440 nm and 490 nm were used to calculate the generated polarization value GP and based on these ΔGP values.

### Tryptophan fluorescence

All experiments were carried out on a Fluoromax-4 (Horiba Scientific) fluorimeter with a peptide concentration of 5 µM in 20 mM Tris pH 7.5, 150 mM NaCl, and the following parameters: excitation wavelength: 280 nm, emission wavelength 300–450 nm, slit width 2 nm, 25 °C.

### Animals

Male B6J;129S8 wild-type (WT) and B6J;129S8 -Trpc6^tm1Lb1^ (MMRRC stock NO 37345-Jax; TRPC6 KO) mice were studied. Animals were housed in groups in standard macrolone cages (Type III). They were provided with food and water *ad libitum*, with paper towels as cage enrichment, and kept on a 12:12 h light:dark cycle (lights on at 0700 hours). All mice were tested at an age of 3–4 months. Behavioral tests were performed during the light cycle between 0900 and 1600 h. Room temperature was maintained between 19 and 22 °C at a humidity of 55% (±10%). All behavioral tests were performed by experimenters blind to hypothesis and/or genotype. All experiments were carried out in accordance with the National Institutes of Health guidelines for the humane treatment of animals and the European Communities Council Directive (86/609/EEC) and approved by the local governmental commission for animal health (German state administration Bavaria/Regierung von Mittelfranken).

### Behavioral testing

Mice were tested in a battery of behavioral tests in the following order: open field, elevated plus maze, light-dark box, novelty suppressed feeding, forced swim test, and sucrose preference tests. All tests were performed on separate days with 3 days between single tests. Mice were tested in a pseudorandom order and were moved to the behavioral suite adjacent to the housing room immediately before testing. Each test apparatus was cleaned with 50% ethanol between subjects to avoid any olfactory cues influencing behaviors. Mice were returned to their home cages at the end of each test and allowed to recover. Only animals that showed a good health during the whole test time and from which a complete data set for each respective paradigm could be measured were analyzed. Behaviors for all tests were recorded on videotape for subsequent scoring by an observer blind to hypothesis and treatment. The behavioral experiments were performed by experimenters who only received animal numbers and test protocols at the time of testing. In the test protocols, order of testing and test box allocation were balanced in a pseudo-random way by the PI. The treatment allocation (randomization) for mice in all animal studies was done by drawing the animal numbers from a bin. Treatment allocation to the mutant and WT mice was done randomly by drawing animal numbers.

#### Open field

Each mouse was placed in a square white acrylic arena (50 × 50 cm), facing an outer wall, for 20 min (parameters were measured per 5-min blocks and summarized) and allowed to freely explore the arena. White light of 25 lx was evenly distributed across the arena during testing. Video recordings were taken and analyzed using Biobserve Viewer III (Biobserve, Bonn, Germany). A virtual square of equal distance from the periphery (36 × 36 cm) was defined as the ‘central zone’ to determine the number of entries and time (s) spent in the central zone. Distance moved in the outer and central zones (cm), number of entries, and time spent in the central zone were registered [63–65].

#### Elevated plus maze

The elevated plus mare was constructed from black opaque acrylic with white lining on the floor, each arm measuring 30 × 5 cm and the central platform 5 × 5 cm. One set of arms, opposing one another, was enclosed completely by a wall of opaque acrylic, 15 cm high, whereas the other set was open with a ledge of 0.5 cm either side of the arms. The maze was elevated 50 cm from the ground on a transparent acrylic stand. Each mouse was placed on the central platform, facing toward a closed arm, and allowed to freely explore the maze for 5 min. Biobserve Viewer III tracking software (Biobserve) was used to record locomotor activity during the test (distance moved in the open and closed arms), and the number of entries into the closed and open arms and time spent in them. An arm entry was counted when two paws had entered an arm, and an arm exit was determined when two paws had left the arm [63–65].

#### Forced swim test

For the forced swim test, each mouse was placed into a glass transparent cylinder (17-cm diameter, 18-cm height) filled with water (12 cm, 25 °C) for 15 min. Then, an animal was returned to the home cage. After 24 h, mice were again placed in this cylinder with water for 5 min. The latency of first floating and total floating time were recorded manually [63–65].

#### Sucrose preference test

Animals were single housed and had access to two bottles with water 7 days before the sucrose preference test. On day 8, water in one bottle was replaced by 2% sucrose solution, and the position of bottles with water and sucrose solution was changed daily during the next 5 days. The weight of animals was measured before and after the test, and volume of water and sucrose solution was estimated daily. Sucrose preference in % of drunken fluid during baseline and testing was calculated [26–28].

### Application of Hyp13 in animals

Hyp13 was dissolved in saline. Animals received an i.p. injection (*v*_inj_ = 10 mL/kg) 20 min before each behavioral test with either, 0, 1, or 5 mg/kg.

### Electrophysiological recordings from brain slices

Horizontal hippocampal slices (350 µm) were prepared from sevoflurane-anesthetized WT and TRPC6-KO male mice (2–4month-old) in ice-cold artificial cerebrospinal fluid (aCSF) containing a high concentration of sucrose (in mM): 75 sucrose, 87 NaCl, 2.5 KCl, 0.5 CaCl_2_, 7 MgCl_2_, 1.25 NaH_2_PO_4_, 25 NaHCO_3_, and 10 D-glucose. Slices were then transferred into warm aCSF (35 °C; 10 min) and kept thereafter at room temperature in aCSF with 125 mM NaCl, 3 mM KCl, 1 mM CaCl_2_, 3 mM MgCl_2_, 1.25 mM NaH_2_PO_4_, 25 mM NaHCO_3_ and 10 mM D-glucose. Individual slices were transferred to a submerged recording chamber (perfused with normal aCSF containing 2.5 mM CaCl_2_ and 1.5 mM MgCl_2_ at 31 ± 1 °C) that was mounted on the stage of an upright microscope. All solutions were gassed with 95% O_2_/5% CO_2_ to keep pH around 7.4. All procedures were conducted in accordance with the Animal Protection Law of Germany and the European Communities Council Directive of November 1986 /86/609/EEC, and with approval of local government.

Hippocampal CA1 pyramidal cells and dentate gyrus (DG) granule cells (in the suprapyramidal blade) were visualized by means of Dodt gradient contrast and recorded with patch pipettes filled with (in mM) 135 K-gluconate, 4 NaCl, 10 KCl, 5 Hepes, 2 Na_2_-ATP and 0.3 Na_3_-GTP (pH 7.3), unless otherwise stated. All potentials were corrected for liquid junction potential. The passive membrane properties of hippocampal neurons (e.g. capacitance, input resistance) were registered from membrane test shortly after breaking into whole-cell mode with membrane voltage clamped at −70 mV. To test neuronal excitability in current-clamp mode, a depolarizing ramp protocol with current injection from 0 to 100 pA within 2 s was used to elicit action potentials (APs) at resting membrane potential (RMP) and at −70 mV. To explore the ionic mechanism of hyperforin-evoked response, granule cells were recorded in voltage-clamp mode (Vh of −70 mV) and a voltage ramp ranging from − 50 to −140 mV (in 1 s) was used to determine the reversal potential. In some experiments, K-gluconate in the pipette solution was replaced by Cs-gluconate (CsGlu).

Data were collected with a Multiclamp 700B amplifier in conjunction with Digidata 1440A interface and pClamp10.2 software (Molecular Devices, Sunnyvale, CA). Signals were digitized at 20 kHz and filtered at 6 kHz (current-clamp) or 2 kHz (voltage-clamp). MiniDigi 1A and AxoScope 10.2 were used for low-resolution scope recording, sampled at 1 kHz. Data analysis was performed offline with Clampfit 10.2 software (Molecular Devices). OriginPro 2018G (OriginLab Corporation, Northampton, MA, USA) was used for statistics and figures.

### Cultivation of HepG2 and HepaRG cells

HepaRG cells (Biopredic International, Sant Grégoire, France) were cultivated in William’s E medium with 2 mM glutamine (PAN-Biotech, Aidenbach, Germany) supplemented with 10% FBS (FBS Good Forte EU approved; PAN-Biotech, Aidenbach, Germany), 100 U/mL penicillin and 100 µg/mL streptomycin (Capricorn Scientific, Ebsdorfergrund, Germany), 0.05% human insulin (PAN-Biotech, Aidenbach, Germany) and 50 µM hydrocortisone-hemisuccinate (Sigma-Aldrich, Taufkirchen, Germany) [41]. Cells were seeded according to the manufacturer’s instructions (96-well plates: 9000 cells/well, 12-well plates 100,000 cells/well). During proliferation, cells were maintained for 2 weeks in culture medium. Afterward, cells were cultured for two more weeks for differentiation in the above-described culture medium, additionally containing 1.7% DMSO. 48 h prior to incubation, HepaRG cells were adapted to treatment medium containing lower concentrations of DMSO (0.5%) and FBS (2%). DMSO concentration has to be reduced to enable the cells to lower their CYP expression. Otherwise, no additional CYP induction will be visible.

Afterward, treatment with the respective test compounds and controls diluted in treatment medium with a final DMSO concentration of 0.5% was performed. HepaRG cells were used for gene expression analysis. Due to poor transfection efficiency of HepaRG cells, reporter gene assays were performed in HepG2 cell.

HepG2 cells (ATCC, Middlesex, UK) were cultivated in DMEM High Glucose medium (Pan-Biotech, Aidenbach, Germany) supplemented with 10% FBS (FBS Superior; Sigma-Aldrich, Taufkirchen, Germany) and 100 U/mL penicillin and 100 µg/mL streptomycin (Capricorn Scientific, Ebsdorfergrund, Germany). Cells were seeded at a confluence of about 80–90% in 96-well plates for experiments.

### Cell viability analysis

Cytotoxic effects of test compounds were analyzed using the cell proliferation reagent WST-1 (Sigma Aldrich, St. Louis, USA). HepG2 cells were seeded in 96-well plates (20,000 cells/well) and were treated with the test compounds 24 h after seeding. HepaRG were seeded in 96-well plates (9000 cells/well) and after 28 days of differentiation and 48 h prior incubation cells were adapted to treatment medium, as described above and afterwards incubated with test compounds for 24 h.

All test compounds and controls are dissolved in culture medium with a final solvent concentration of 0.5% DMSO. Triton X-100 (0.01%) served as a positive control. One hour before incubation ends, 10 μL WTS-1 reagent was added to each well containing 100 μL medium, and plates were incubated one more hour. Afterwards, absorbance was measured at 450 nm with a reference wavelength of 620 nm using the Tecan plate reader Infinite M200 Pro (Tecan group, Männedorf, Switzerland). Values of the reference wavelength were subtracted from absorbance values, and data were corrected for background absorbance by subtracting the values from wells incubated without cells. Data were referred to solvent control, which was set to 100%. At least three independent, biological replicates with three technical replicates per condition were performed.

### Dual luciferase reporter assay

Dual luciferase reporter assays were conducted to measure the activation of *human pregnane X receptor (PXR)* via a GAL4-based transactivation assay, for which the LBD of *PXR* had been fused to the DBD of GAL4 (pGAL4-h*PXR*-LBD), coupled with a GAL4-responsive UAS-driven firefly luciferase reporter (pGAL4-(UAS)5-TK-LUC) [40]. Agonistic binding of a test compound to the LBD of *PXR* leads to the activation of the fusion protein, which binds to the UAS and initiates transcription of the firefly luciferase gene.

Additionally, cells were always transfected with a plasmid constitutively expressing the reporter gene Renilla luciferase (pcDNA3-Rluc) as internal control for normalization. Plasmid concentrations and positive controls are listed in Table 1.

**Table 1:** Plasmids used for dual luciferase reporter assays in HepG2 cells.

Reporter gene assay	Plasmid	Plasmid control	Positive control
PXR (24 h)	pGAL4-(UAS)5-TK-LUC	40 ng/well	10 µM SR12813 /
	pGAL4-PXR-LBD	40 ng/well	10 µM Rifampicin
	pcDNA3-Rluc	1 ng/well	

To perform dual luciferase reporter assays 20´000 HepG2 cells were seeded in 96-well plates for the *PXR* assay. Cell were transiently transfected 24 h after seeding using TransIT-LT1 (Mirus Bio LCC, Madison, WI, USA) according to manufacturer´s protocol. After 4–6 h, cells were incubated with test compounds or controls in culture medium containing 0.5% DMSO. As positive controls, the well-known *PXR* agonists SR12813 and Rifampicin were used. After 24 h of incubation, cells were lysed with 50 µL lysis buffer (100 mM potassium phosphate with 0.2% (v/v) Triton X-100, pH 7.8). Five µL cell lysate was investigated for firefly and Renilla luciferase activities. Luminescence was measured with an Infinite M200 Pro plate reader (Tecan group, Männedorf, Switzerland). The firefly signal was normalized to the Renilla signal and was expressed relative to the solvent control (containing 0.5% DMSO). Three independent, biological replicates were performed with six technical replicates per condition.

### Gene expression analysis

HepaRG cells were seeded in 12-well plates (100,000 cells/wells) and cultivated as described above. After differentiation of the cells followed by 48 h of cultivation with treatment medium, cells were incubated for 24 h with test compounds and Rifampicin as a positive control. Cells were washed twice with ice-cold PBS and harvested with 350 μL RLT buffer (RNeasy Mini Kit, Qiagen, Hilden Germany) containing 3.5 μL β-mercaptoethanol. Total RNA was extracted following the manufacturer’s instructions with minor modifications [66]. RNA quality and quantity were measured with a Tecan Infinite M200 Pro plate reader (Tecan group, Männedorf, Switzerland). Reverse transcription of 0.5 µg RNA was done with the High-Capacity cDNA Reverse Transcription Kit (Applied Biosystems, Darmstadt, Germany). Quantitative real-time RT-PCR was performed using 20 ng cDNA, the Maxima SYBR Green/ROX qPCR Master Mix (Thermofisher Scientific, Waltham, Massachusetts, USA) and primers (5 µM; see Table 2). Primer design was performed by using the free available software tool Primer3 (University of Massachusetts Medical School, USA). Primers were designed intron-spanning and checked for mispriming, hairpins and dimers using NetPrimer (University of Massachusetts Medical School, USA) and the ensemble database (www.ensembl.org). Primers were purchased from Eurofins Genomics Germany GmbH (Ebersberg, Germany).

**Table 2:** Sequences of qPCR primers.

Gene	Accession number	forward primer (5′− 3′)	reverse primer (5′− 3′)
CYP3A4	ENSG00000160868	TCAGCCTGGTGCTCCTCTATCTAT	AAGCCCTTATGGTAGGACAAAATATTT
GAPDH	ENSG00000111640	TTAAAAGCAGCCCTGGTGAC	CTCTGCTCCTCCTGTTCGAC
ACTB	ENSG00000075624	CCTTGCACATGCCGGAG	GCACAGAGCCTCGCCTT

Gene expression was measured with a Stratagene MX3005P real-time PCR cycler (Agilent Technologies, Santa Clara, CA, USA). Expression levels of the target gene *CYP3A4* were normalized to the geometric mean of the reference genes ACTB and GAPDH, which were found to be stably expressed throughout treatments. RNA from three independent, biological replicates was used. Relative gene expression was calculated using the ΔΔCT method. Three independent, biological replicates were performed, outliers were identified via Grubb´s test and excluded from the analysis.

### Statistical analyses

Statistical analyses were performed using GraphPad Prism 7 or GraphPad Prism 8 (Graphpad Software, LaJolla, CA, USA). Data are shown as mean ± standard error of the means (SEM) or (SD). For behavioral and neurochemical animal studies, sample sizes were chosen to obtain an effect size of Cohen *d* > 0.5 with *α* = 5% and a power of 0.8. ample size for animal studies was determined by previous experience with those test paradigms and mean values and variance derived therefrom. This was fed into a G-Power analysis for the main read out of each paradigm. Data distribution was checked with D’Agostino Pearson omnibus normality test. Unpaired student’s *t*-test or Mann-Whitney test were used if experiments consisted of two data sets. For considering two different parameters, two-way ANOVA was used with Sidak’s multiple comparison test. *p* ≤ 0.05 was considered as statistically significant.

## Supplementary information

Supplementary Information

## Data Availability

The data that support the findings of this study are available from the corresponding author upon request.

## References

[1] Vigo D, Thornicroft G, Atun R (2016). Estimating the true global burden of mental illness. Lancet Psychiatry.

[2] Park LT, Zarate CA (2019). Depression in the primary care setting. N Engl J Med.

[3] Duman RS, Aghajanian GK, Sanacora G, Krystal JH (2016). Synaptic plasticity and depression: New insights from stress and rapid-acting antidepressants. Nat Med.

[4] Linde K, Mulrow CD (2000). St John’s wort for depression. Cochrane Database Syst Rev.

[5] Linde K, Berner MM, Kriston L (2008). St John’s wort for major depression. Cochrane Database Syst Rev.

[6] Apaydin EA, Maher AR, Shanman R, Booth MS, Miles JNV, Sorbero ME (2016). A systematic review of St. John’s wort for major depressive disorder. Syst Rev.

[7] Friedland K, Harteneck C (2015). Hyperforin: To be or not to be an activator of TRPC(6). Rev Physiol Biochem Pharmacol.

[8] Sell TS, Belkacemi T, Flockerzi V, Beck A (2014). Protonophore properties of hyperforin are essential for its pharmacological activity. Sci Rep.

[9] Leuner K, Kazanski V, Müller M, Essin K, Henke B, Gollasch M (2007). Hyperforin-a key constituent of St. John’s wort specifically activates TRPC6 channels. FASEB J.

[10] Lu R, Wang J, Tao R, Wang J, Zhu T, Guo W (2018). Reduced TRPC6 mRNA levels in the blood cells of patients with Alzheimer’s disease and mild cognitive impairment. Mol Psychiatry.

[11] Hellmich UA, Gaudet R (2014). Structural biology of TRP channels. Handb Exp Pharmacol.

[12] Vangeel L, Voets T (2019). Transient receptor potential channels and calcium signaling. Cold Spring Harb Perspect Biol.

[13] Goretzki B, Glogowski NA, Diehl E, Duchardt-Ferner E, Hacker C, Gaudet R (2018). Structural basis of TRPV4 N terminus interaction with Syndapin/PACSIN1-3 and PIP 2. Structure..

[14] Hellmich UA, Gaudet R (2014). High-resolution views of TRPV1 and their implications for the TRP channel superfamily. Handb Exp Pharmacol.

[15] Wang H, Cheng X, Tian J, Xiao Y, Tian T, Xu F (2020). TRPC channels: structure, function, regulation and recent advances in small molecular probes. Pharmacol Ther.

[16] Goretzki B, Guhl C, Tebbe F, Harder JM, Hellmich UA (2021). Unstructural biology of TRP ion channels: the role of intrinsically disordered regions in channel function and regulation. J Mol Biol.

[17] Bacsa B, Tiapko O, Stockner T, Groschner K (2020). Mechanisms and significance of Ca2^+^ entry through TRPC channels. Curr Opin Physiol.

[18] Li J, Zhang X, Song X, Liu R, Zhang J, Li Z (2019). The structure of TRPC ion channels. Cell Calcium.

[19] Bai Y, Yu X, Chen H, Horne D, White R, Wu X (2020). Structural basis for pharmacological modulation of the TRPC6 channel. Elife.

[20] Azumaya CM, Sierra-Valdez F, Cordero-Morales JF, Nakagawa T (2018). Cryo-EM structure of the cytoplasmic domain of murine transient receptor potential cation channel subfamily C member 6 (TRPC6). J Biol Chem.

[21] Fan C, Choi W, Sun W, Du J, Lu W (2018). Structure of the human lipid-gated cation channel TRPC3. Elife.

[22] Tang Q, Guo W, Zheng L, Wu JX, Liu M, Zhou X (2018). Structure of the receptor-activated human TRPC6 and TRPC3 ion channels. Cell Res.

[23] Nagy GA, Botond G, Borhegyi Z, Plummer NW, Freund TF, Hájos N (2013). DAG-sensitive and Ca^2+^ permeable TRPC6 channels are expressed in dentate granule cells and interneurons in the hippocampal formation. Hippocampus..

[24] Leuner K, Li W, Amaral MD, Rudolph S, Calfa G, Schuwald AM (2013). Hyperforin modulates dendritic spine morphology in hippocampal pyramidal neurons by activating Ca(^2+^) -permeable TRPC6 channels. Hippocampus..

[25] Li Y, Jin YC, Cul K, Li N, Zheng ZY, Wang YZ (2005). Essential role of TRPC channels in the guidance of nerve growth cones by brain-derived neurotrophic factor. Nature.

[26] Zhou J, Du W, Zhou K, Tai Y, Yao H, Jia Y (2008). Critical role of TRPC6 channels in the formation of excitatory synapses. Nat Neurosci.

[27] Heiser JH, Schuwald AM, Sillani G, Ye L, Müller WE, Leuner K (2013). TRPC6 channel-mediated neurite outgrowth in PC12 cells and hippocampal neurons involves activation of RAS/MEK/ERK, PI3K, and CAMKIV signaling. J Neurochem.

[28] Li H, Huang J, Du W, Jia C, Yao H, Wang Y (2012). TRPC6 inhibited NMDA receptor activities and protected neurons from ischemic excitotoxicity. J Neurochem.

[29] Leuner K, Heiser JH, Derksen S, Mladenov MI, Fehske CJ, Schubert R (2010). Simple 2,4-diacylphloroglucinols as classic transient receptor potential-6 activators-identification of a novel pharmacophore. Mol Pharmacol.

[30] Kandel BA, Ekins S, Leuner K, Thasler WE, Harteneck C, Zanger UM (2014). No activation of human pregnane X receptor by hyperforin-related phloroglucinols. J Pharmacol Exp Ther.

[31] Dietrich A, Mederos y Schnitzler M, Gollasch M, Gross V, Storch U, Dubrovska G (2005). Increased Vascular Smooth Muscle Contractility in TRPC6 − / − Mice. Mol Cell Biol.

[32] Malberg JE, Hen R, Madsen TM (2021). Adult neurogenesis and antidepressant treatment: the surprise finding by Ron Duman and the field 20 years later. Biol Psychiatry.

[33] Hare BD, Duman RS (2020). Prefrontal cortex circuits in depression and anxiety: contribution of discrete neuronal populations and target regions. Mol Psychiatry.

[34] Gulbins A, Schumacher F, Becker KA, Wilker B, Soddemann M, Boldrin F (2018). Antidepressants act by inducing autophagy controlled by sphingomyelin–ceramide. Mol Psychiatry.

[35] Kalinichenko LS, Mühle C, Jia T, Anderheiden F, Datz M, Eberle A-L (2021). Neutral sphingomyelinase mediates the co-morbidity trias of alcohol abuse, major depression and bone defects. Mol Psychiatry.

[36] Gulbins E, Palmada M, Reichel M, Lüth A, Böhmer C, Amato D (2013). Acid sphingomyelinase-ceramide system mediates effects of antidepressant drugs. Nat Med.

[37] Chatterjee S, Filippov V, Lishko P, Maximyuk O, Nöldner M, Krishtal O (1999). Hyperforin attenuates various ionic conductance mechanisms in the isolated hippocampal neurons of rat. Life Sci.

[38] Hofmann T, Obukhov AG, Schaefer M, Harteneck C, Gudermann T, Schultz G (1999). Direct activation of human TRPC6 and TRPC3 channels by diacylglycerol. Nature..

[39] Parasassi T, Gratton E (1995). Membrane lipid domains and dynamics as detected by Laurdan fluorescence. J Fluoresc.

[40] Luckert C, Ehlers A, Buhrke T, Seidel A, Lampen A, Hessel S (2013). Polycyclic aromatic hydrocarbons stimulate human CYP3A4 promoter activity via PXR. Toxicol Lett.

[41] Luckert C, Braeuning A, De Sousa G, Durinck S, Katsanou ES, Konstantinidou P (2018). Adverse outcome pathway-driven analysis of liver steatosis in vitro: a case study with cyproconazole. Chem Res Toxicol.

[42] Liu Y, Liu C, Qin X, Zhu M, Yang Z (2015). The change of spatial cognition ability in depression rat model and the possible association with down-regulated protein expression of TRPC6. Behav Brain Res.

[43] Xie R, Wang Z, Liu T, Xiao R, Lv K, Wu C (2021). AAV delivery of shRNA against TRPC6 in mouse hippocampus impairs cognitive function. Front Cell Dev Biol.

[44] Beis D, Schwarting RKW, Dietrich A (2011). Evidence for a supportive role of classical transient receptor potential 6 (TRPC6) in the exploration behavior of mice. Physiol Behav.

[45] Griesi-Oliveira K, Acab A, Gupta AR, Sunaga DY, Chailangkarn T, Nicol X (2015). Modeling non-syndromic autism and the impact of TRPC6 disruption in human neurons. Mol Psychiatry.

[46] Lu R, He Q, Wang J (2017). TRPC channels and Alzheimer’s disease. Adv Exp Med Biol.

[47] Licznerski P, Duman RS (2013). Remodeling of axo-spinous synapses in the pathophysiology and treatment of depression. Neuroscience..

[48] Lisman JE, Grace AA (2005). The hippocampal-VTA loop: controlling the entry of information into long-term memory. Neuron..

[49] LeGates TA, Kvarta MD, Tooley JR, Francis TC, Lobo MK, Creed MC (2018). Reward behaviour is regulated by the strength of hippocampus–nucleus accumbens synapses. Nature..

[50] Mertens J, Wang QW, Kim Y, Yu DX, Pham S, Yang B (2015). Differential responses to lithium in hyperexcitable neurons from patients with bipolar disorder. Nature..

[51] Airan RD, Meltzer LA, Roy M, Gong Y, Chen H, Deisseroth K (2007). High-speed imaging reveals neurophysiological links to behavior in an animal model of depression. Science..

[52] Zeitler S, Schumacher F, Monti J, Anni D, Guhathakurta D, Kleuser B (2020). Acid sphingomyelinase impacts canonical transient receptor potential channels 6 (TRPC6) activity in primary neuronal systems. Cells..

[53] Zeitler S, Ye L, Andreyeva A, Schumacher F, Monti J, Nürnberg B (2019). Acid sphingomyelinase – a regulator of canonical transient receptor potential channel 6 (TRPC6) activity. J Neurochem.

[54] Szewczyk B, Pochwat B, Muszyńska B, Opoka W, Krakowska A, Rafało-Ulińska A (2019). Antidepressant-like activity of hyperforin and changes in BDNF and zinc levels in mice exposed to chronic unpredictable mild stress. Behav Brain Res.

[55] Gibon J, Deloulme JC, Chevallier T, Ladevèze E, Abrous DN, Bouron A (2013). The antidepressant hyperforin increases the phosphorylation of CREB and the expression of TrkB in a tissue-specific manner. Int J Neuropsychopharmacol.

[56] Müller WE, Singer A, Wonnemann M (2001). Hyperforin - antidepressant activity by a novel mechanism of action. Pharmacopsychiatry..

[57] Müller WE (2003). Current St. John’s wort research from mode of action to clinical efficacy. Pharmacol Res.

[58] Pochwat B, Szewczyk B, Kotarska K, Rafało-Ulińska A, Siwiec M, Sowa JE (2018). Hyperforin potentiates antidepressant-like activity of lanicemine in mice. Front Mol Neurosci.

[59] Shi J, Geshi N, Takahashi S, Kiyonaka S, Ichikawa J, Hu Y (2013). Molecular determinants for cardiovascular TRPC6 channel regulation by Ca^2+^/calmodulin-dependent kinase II. J Physiol.

[60] Keller JH, Karas M, Müller WE, Volmer DA, Eckert GP, Tawab MA (2003). Determination of hyperforin in mouse brain by high-performance liquid chromatography/tandem mass spectrometry. Anal Chem.

[61] Porsolt RD, Le Pichon M, Jalfre M (1977). Depression: a new animal model sensitive to antidepressant treatments. Nature..

[62] Bharate SB, Rodge A, Joshi RK, Kaur J, Srinivasan S, Senthil Kumar S (2008). Discovery of diacylphloroglucinols as a new class of GPR40 (FFAR1) agonists. Bioorg Med Chem Lett.

[63] Mielenz D, Reichel M, Jia T, Quinlan EB, Stöckl T, Mettang M (2018). EFhd2/Swiprosin-1 is a common genetic determinator for sensation-seeking/low anxiety and alcohol addiction. Mol Psychiatry.

[64] Süß P, Kalinichenko L, Baum W, Reichel M, Kornhuber J, Loskarn S (2015). Hippocampal structure and function are maintained despite severe innate peripheral inflammation. Brain Behav Immun.

[65] Easton AC, Lucchesi W, Schumann G, Peter Giese K, Müller CP, Fernandes C (2011). αcaMKII autophosphorylation controls exploratory activity to threatening novel stimuli. Neuropharmacology..

[66] Lichtenstein D, Mentz A, Schmidt FF, Luckert C, Buhrke T, Marx-Stoelting P (2020). Transcript and protein marker patterns for the identification of steatotic compounds in human HepaRG cells. Food Chem Toxicol.

