# Supplementary material for: Analysis of hyperforin (St. John’s wort) action at TRPC6 channel leads to the development of a new class of antidepressant drugs
Source: Mol Psychiatry. 2022 Oct 12;27(12):5070–85. doi: 10.1038/s41380-022-01804-3 (PMC9763113; doi:10.1038/s41380-022-01804-3)
Supplement: Supplementary file 1 — Supplementary Information [file 41380_2022_1804_MOESM1_ESM.pptx]

## Slide 1
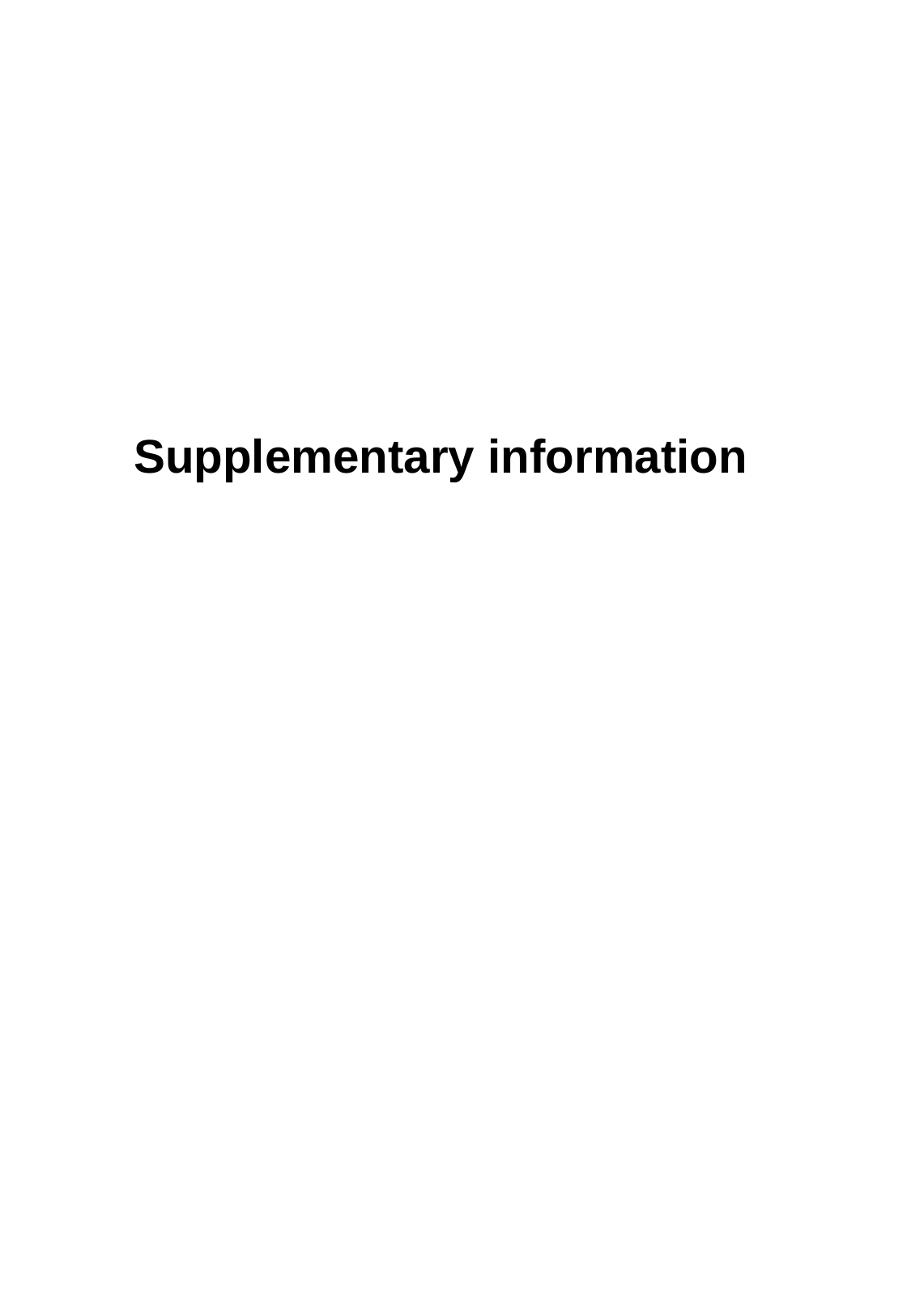

Supplementary information

## Slide 2
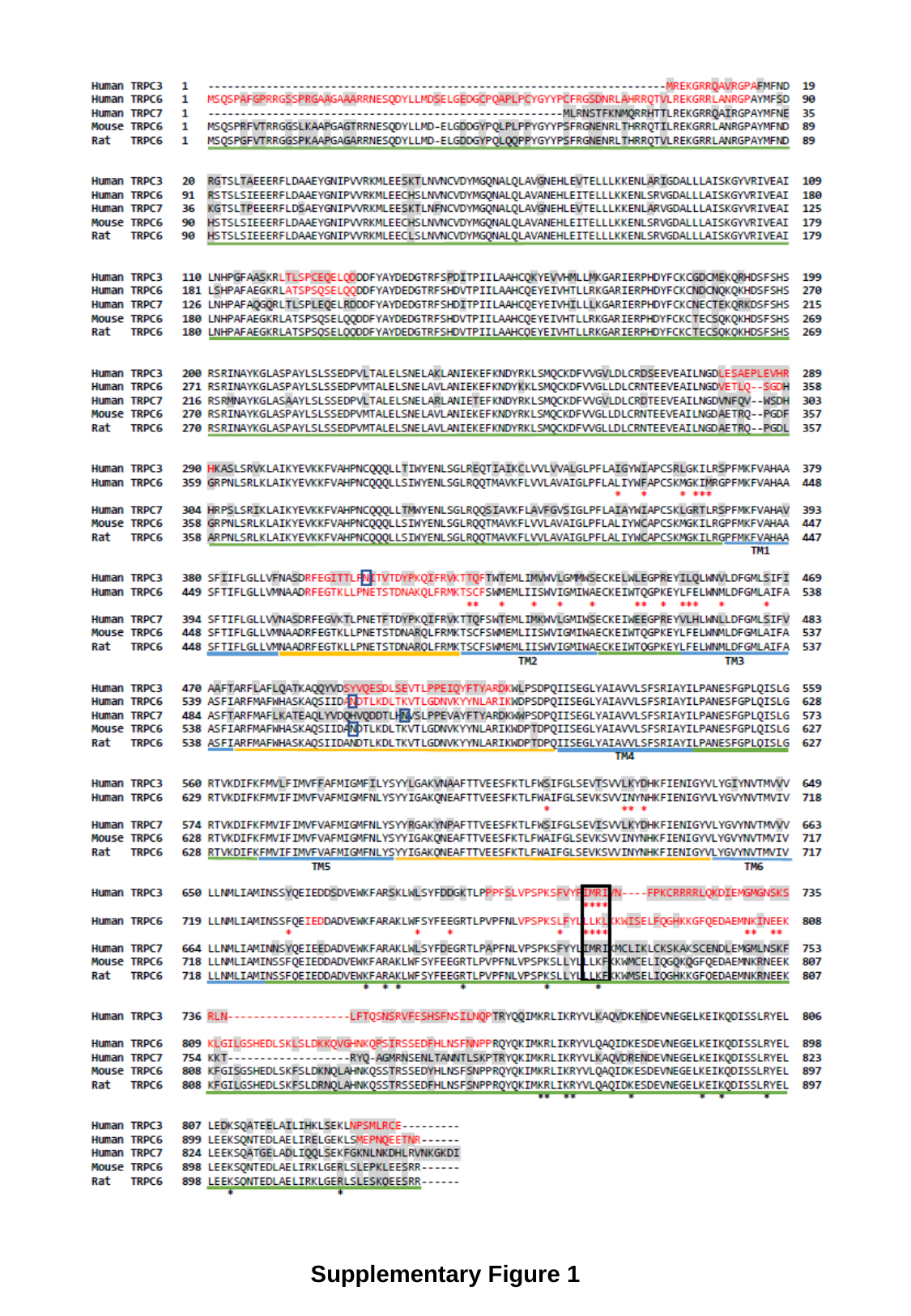

Supplementary Figure 1

## Slide 3
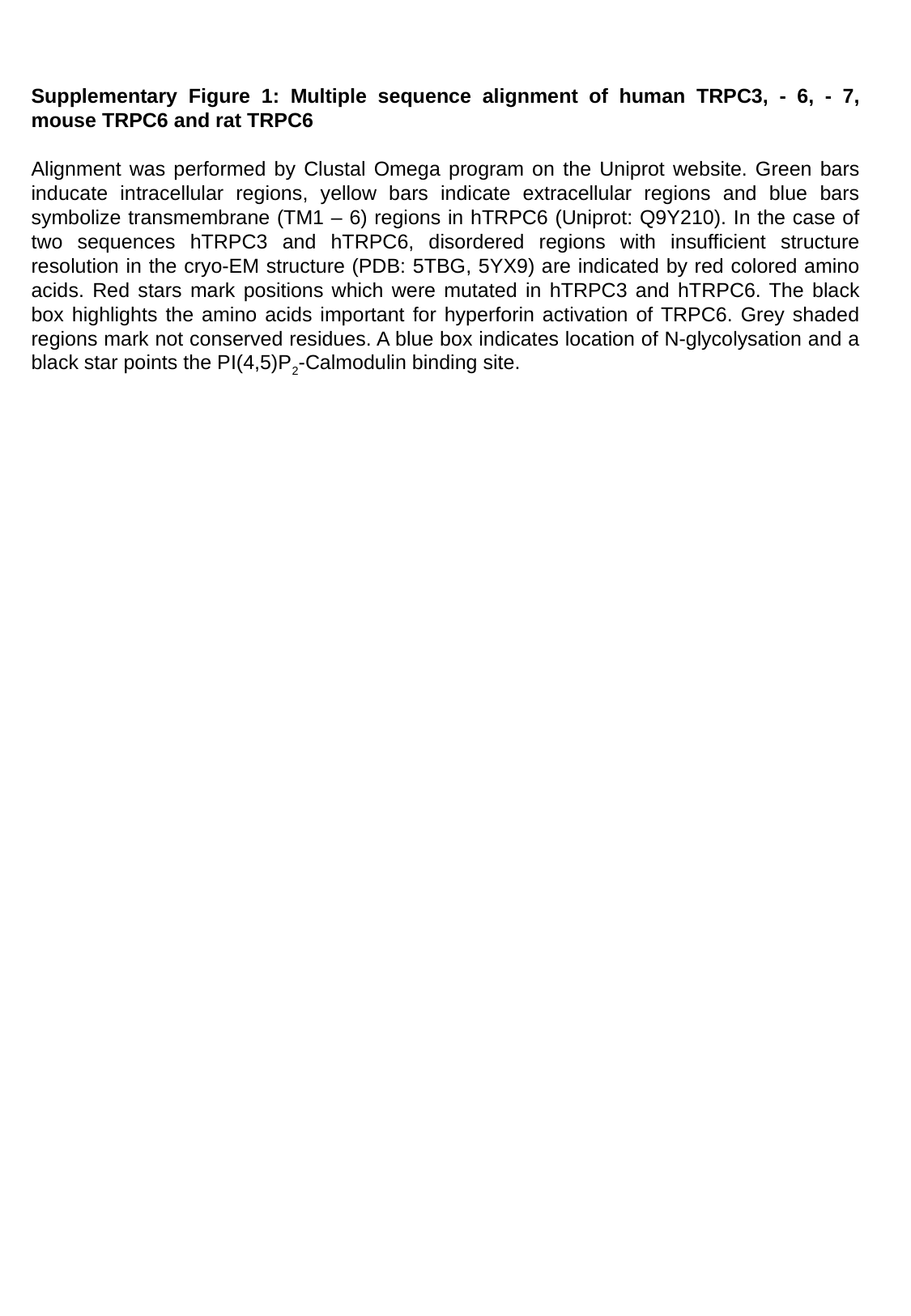

Supplementary Figure 1: Multiple sequence alignment of human TRPC3, - 6, - 7, mouse TRPC6 and rat TRPC6
Alignment was performed by Clustal Omega program on the Uniprot website. Green bars inducate intracellular regions, yellow bars indicate extracellular regions and blue bars symbolize transmembrane (TM1 – 6) regions in hTRPC6 (Uniprot: Q9Y210). In the case of two sequences hTRPC3 and hTRPC6, disordered regions with insufficient structure resolution in the cryo-EM structure (PDB: 5TBG, 5YX9) are indicated by red colored amino acids. Red stars mark positions which were mutated in hTRPC3 and hTRPC6. The black box highlights the amino acids important for hyperforin activation of TRPC6. Grey shaded regions mark not conserved residues. A blue box indicates location of N-glycolysation and a black star points the PI(4,5)P2-Calmodulin binding site.

## Slide 4
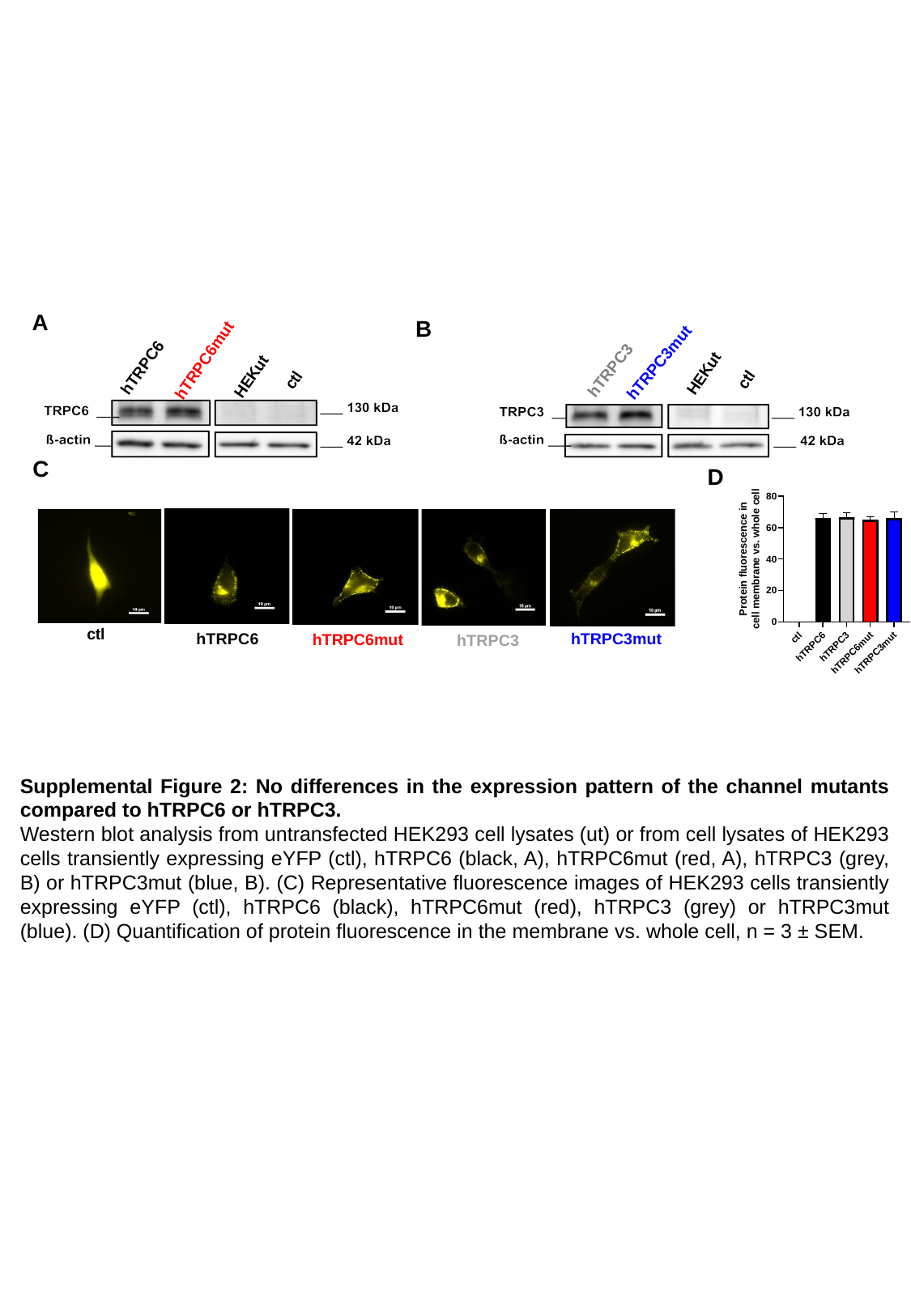

Supplemental Figure 2: No differences in the expression pattern of the channel mutants compared to hTRPC6 or hTRPC3.
Western blot analysis from untransfected HEK293 cell lysates (ut) or from cell lysates of HEK293 cells transiently expressing eYFP (ctl), hTRPC6 (black, A), hTRPC6mut (red, A), hTRPC3 (grey, B) or hTRPC3mut (blue, B). (C) Representative fluorescence images of HEK293 cells transiently expressing eYFP (ctl), hTRPC6 (black), hTRPC6mut (red), hTRPC3 (grey) or hTRPC3mut (blue). (D) Quantification of protein fluorescence in the membrane vs. whole cell, n = 3 ± SEM.

## Slide 5
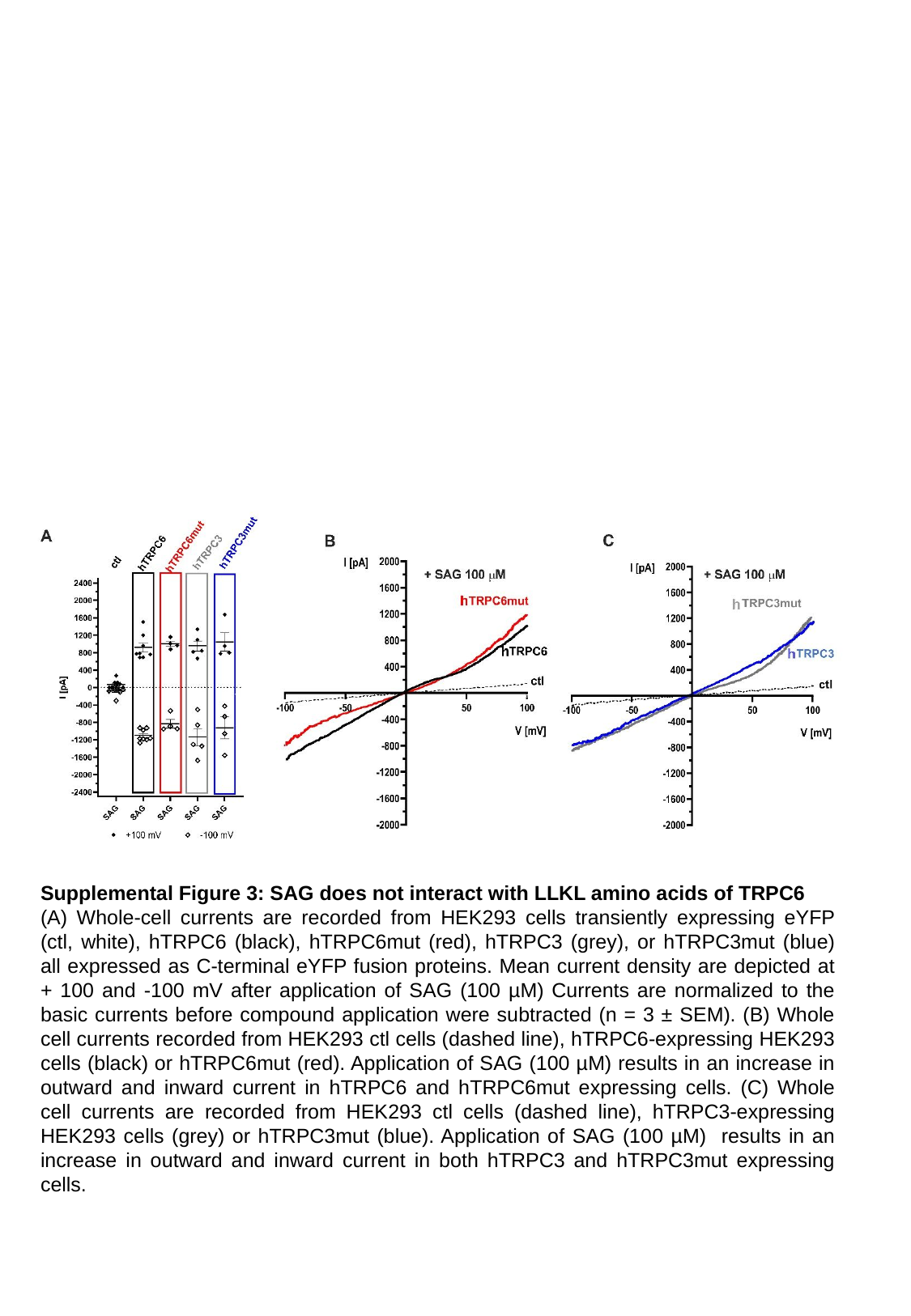

Supplemental Figure 3: SAG does not interact with LLKL amino acids of TRPC6
(A) Whole-cell currents are recorded from HEK293 cells transiently expressing eYFP (ctl, white), hTRPC6 (black), hTRPC6mut (red), hTRPC3 (grey), or hTRPC3mut (blue) all expressed as C-terminal eYFP fusion proteins. Mean current density are depicted at + 100 and -100 mV after application of SAG (100 µM) Currents are normalized to the basic currents before compound application were subtracted (n = 3 ± SEM). (B) Whole cell currents recorded from HEK293 ctl cells (dashed line), hTRPC6-expressing HEK293 cells (black) or hTRPC6mut (red). Application of SAG (100 µM) results in an increase in outward and inward current in hTRPC6 and hTRPC6mut expressing cells. (C) Whole cell currents are recorded from HEK293 ctl cells (dashed line), hTRPC3-expressing HEK293 cells (grey) or hTRPC3mut (blue). Application of SAG (100 µM) results in an increase in outward and inward current in both hTRPC3 and hTRPC3mut expressing cells.

## Slide 6
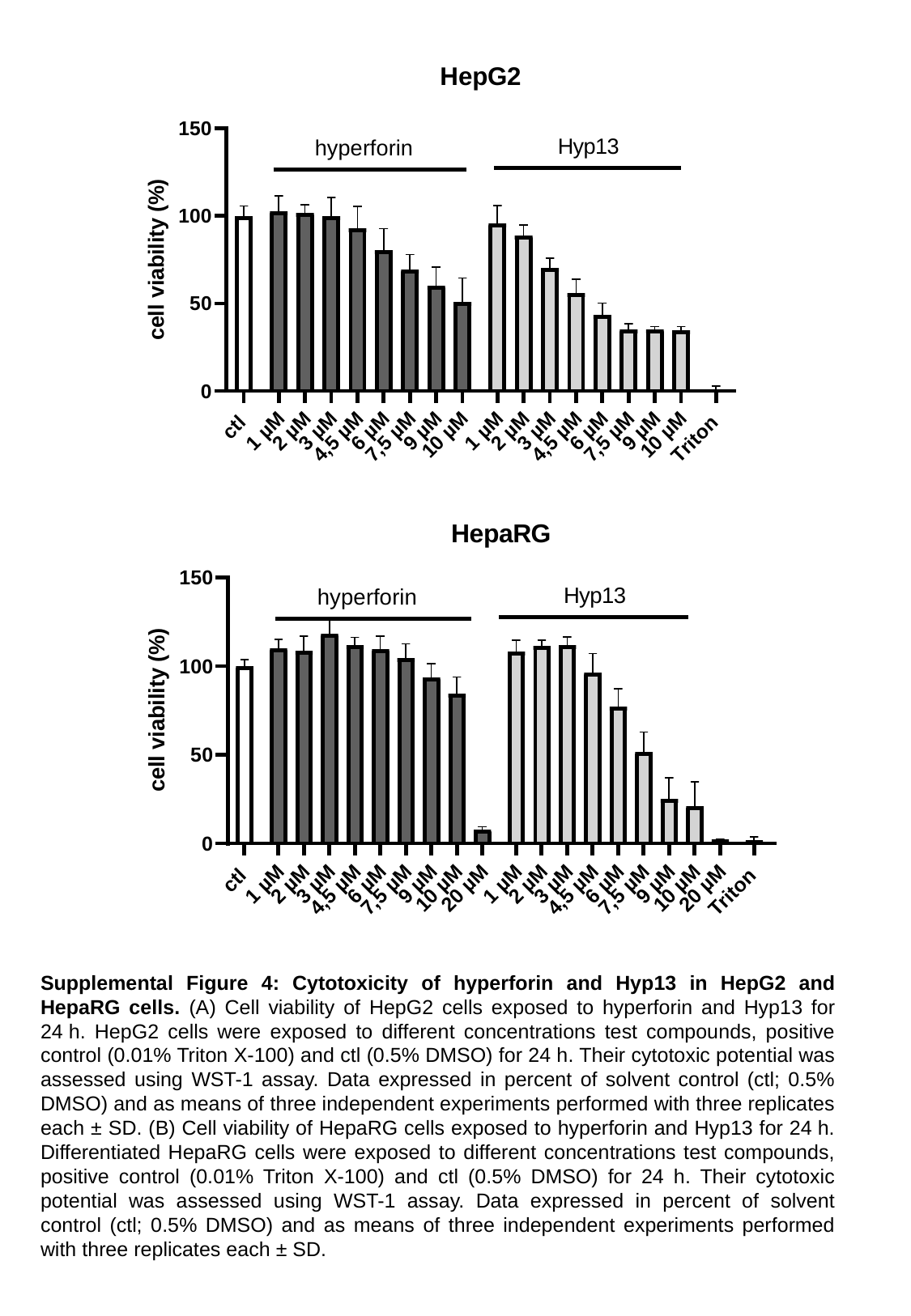

Supplemental Figure 4: Cytotoxicity of hyperforin and Hyp13 in HepG2 and HepaRG cells. (A) Cell viability of HepG2 cells exposed to hyperforin and Hyp13 for 24 h. HepG2 cells were exposed to different concentrations test compounds, positive control (0.01% Triton X-100) and ctl (0.5% DMSO) for 24 h. Their cytotoxic potential was assessed using WST-1 assay. Data expressed in percent of solvent control (ctl; 0.5% DMSO) and as means of three independent experiments performed with three replicates each ± SD. (B) Cell viability of HepaRG cells exposed to hyperforin and Hyp13 for 24 h. Differentiated HepaRG cells were exposed to different concentrations test compounds, positive control (0.01% Triton X-100) and ctl (0.5% DMSO) for 24 h. Their cytotoxic potential was assessed using WST-1 assay. Data expressed in percent of solvent control (ctl; 0.5% DMSO) and as means of three independent experiments performed with three replicates each ± SD.

## Slide 7
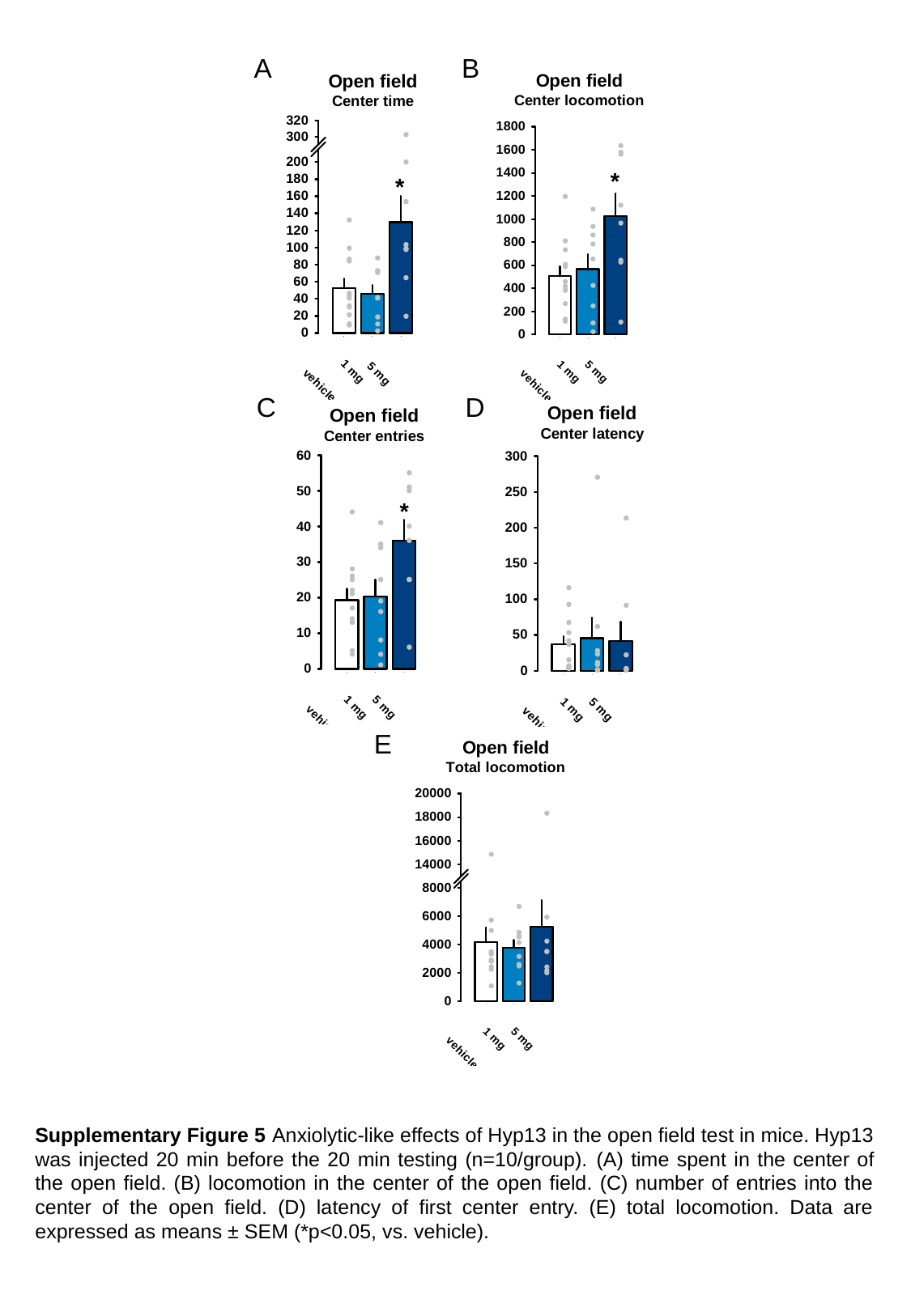

A
B
*
*
C
D
*
E
Supplementary Figure 5 Anxiolytic-like effects of Hyp13 in the open field test in mice. Hyp13 was injected 20 min before the 20 min testing (n=10/group). (A) time spent in the center of the open field. (B) locomotion in the center of the open field. (C) number of entries into the center of the open field. (D) latency of first center entry. (E) total locomotion. Data are expressed as means ± SEM (*p<0.05, vs. vehicle).

## Slide 8
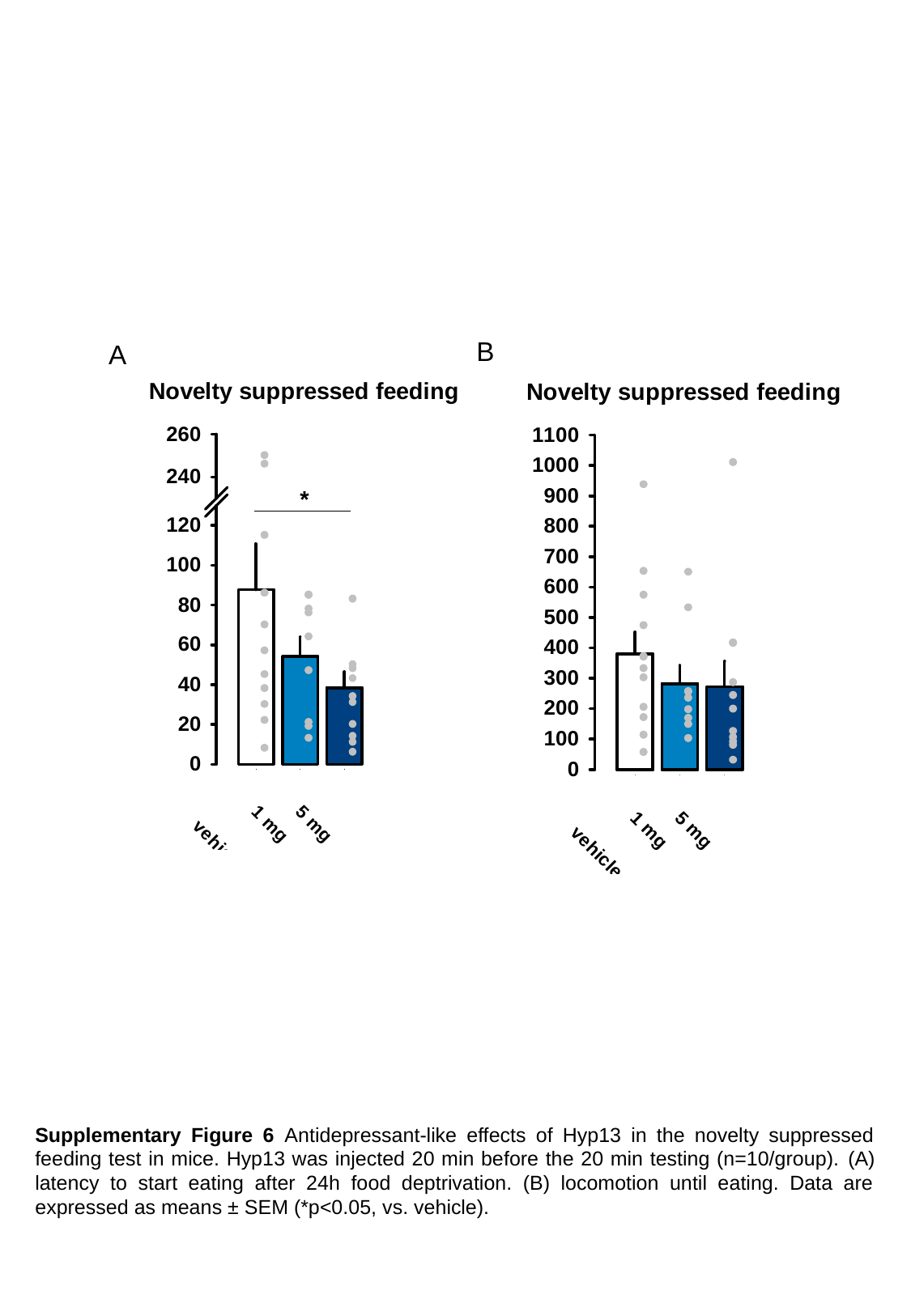

B
A
*
Supplementary Figure 6 Antidepressant-like effects of Hyp13 in the novelty suppressed feeding test in mice. Hyp13 was injected 20 min before the 20 min testing (n=10/group). (A) latency to start eating after 24h food deptrivation. (B) locomotion until eating. Data are expressed as means ± SEM (*p<0.05, vs. vehicle).

## Slide 9
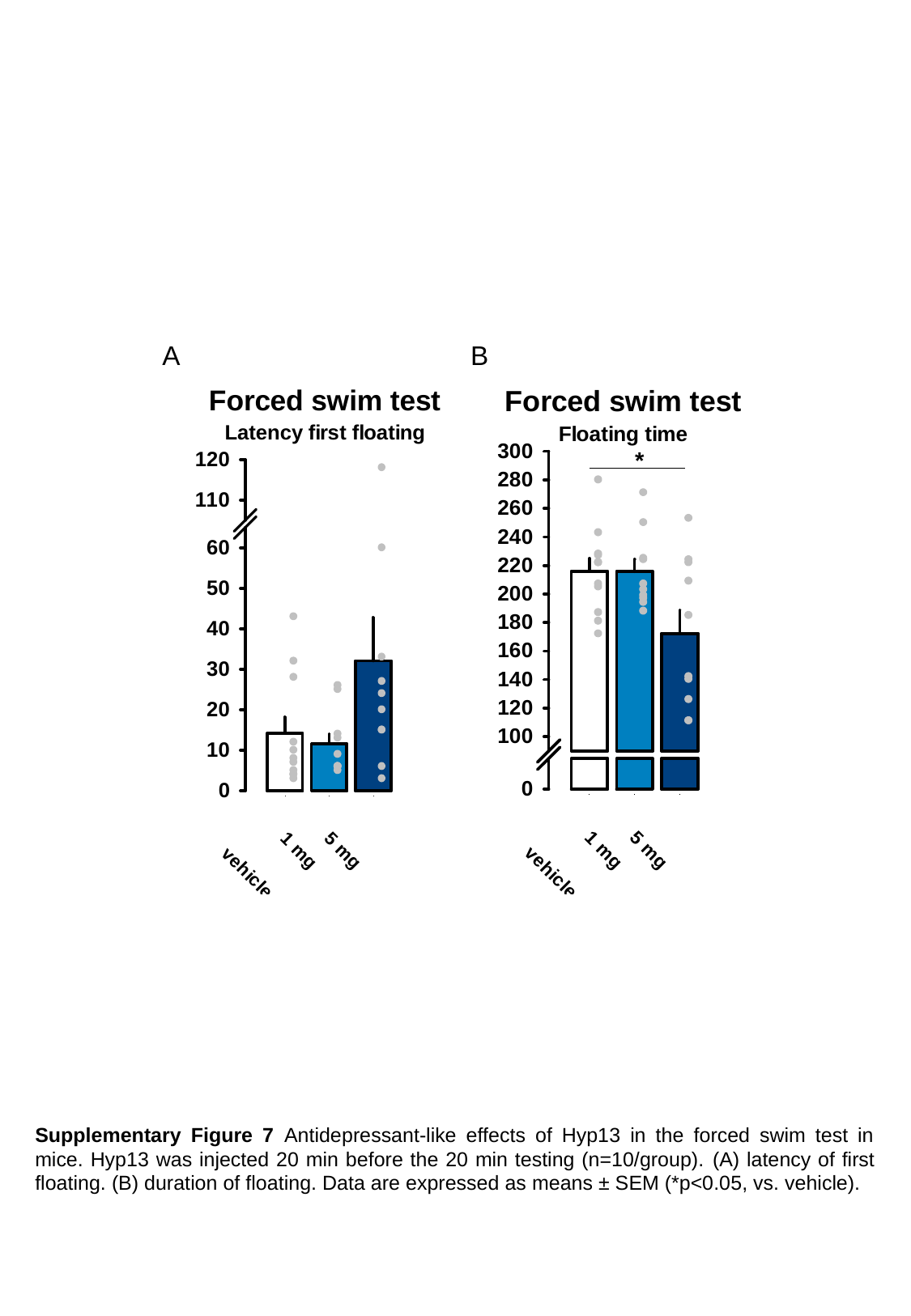

A
B
*
Supplementary Figure 7 Antidepressant-like effects of Hyp13 in the forced swim test in mice. Hyp13 was injected 20 min before the 20 min testing (n=10/group). (A) latency of first floating. (B) duration of floating. Data are expressed as means ± SEM (*p<0.05, vs. vehicle).
